# Overview and Prospects of DNA Sequence Visualization

**DOI:** 10.3390/ijms26020477

**Published:** 2025-01-08

**Authors:** Yan Wu, Xiaojun Xie, Jihong Zhu, Lixin Guan, Mengshan Li

**Affiliations:** School of Mathematics and Computer Science, Gannan Normal University, Ganzhou 341000, China; wuyan@gnnu.edu.cn (Y.W.); xiexiaojun@gnnu.edu.cn (X.X.); zhujihong@gnnu.edu.cn (J.Z.); lxguan@gnnu.edu.cn (L.G.)

**Keywords:** DNA sequences, biological sequence, visualization, knowledge graph, machine learning

## Abstract

Due to advances in big data technology, deep learning, and knowledge engineering, biological sequence visualization has been extensively explored. In the post-genome era, biological sequence visualization enables the visual representation of both structured and unstructured biological sequence data. However, a universal visualization method for all types of sequences has not been reported. Biological sequence data are rapidly expanding exponentially and the acquisition, extraction, fusion, and inference of knowledge from biological sequences are critical supporting technologies for visualization research. These areas are important and require in-depth exploration. This paper elaborates on a comprehensive overview of visualization methods for DNA sequences from four different perspectives—two-dimensional, three-dimensional, four-dimensional, and dynamic visualization approaches—and discusses the strengths and limitations of each method in detail. Furthermore, this paper proposes two potential future research directions for biological sequence visualization in response to the challenges of inefficient graphical feature extraction and knowledge association network generation in existing methods. The first direction is the construction of knowledge graphs for biological sequence big data, and the second direction is the cross-modal visualization of biological sequences using machine learning methods. This review is anticipated to provide valuable insights and contributions to computational biology, bioinformatics, genomic computing, genetic breeding, evolutionary analysis, and other related disciplines in the fields of biology, medicine, chemistry, statistics, and computing. It has an important reference value in biological sequence recommendation systems and knowledge question answering systems.

## 1. Introduction

### 1.1. Background

With the advent of the big data era, the field of life sciences has entered the post-genomic era characterized by “omics”. The Human Genome Project has transitioned and transformed from structural genomics to functional genomics [1,2,3,4]. Research on gene expression and its regulation, genomic diversity, and model organism genomes has become the core issue [5,6]. The development of sequencing technologies has provided support for obtaining massive amounts of sequencing data. However, the continuous increase in data volume has brought forth a new challenge of how to store, manage, and analyze data [7,8,9]. Data are not equivalent to knowledge. The interpretation and application of data have become crucial tasks for the future. High-throughput sequencing data are stored in textual format, which presents characteristics such as being unstructured, multi-source, heterogeneous, and difficult to integrate and utilize due to its high specialization [10,11]. The human brain struggles to directly extract knowledge from textual data, which is why scholars have long been committed to extracting knowledge from textual data through visualization. The traditional representation of DNA sequences is through the use of the Letter Sequence Representation (LSR) method [12]. LSR has made significant contributions to the storage and statistical analysis of sequence information. However, it is often challenging to extract knowledge and analyze sequence features in LSR. Especially for long sequences, it can be difficult to intuitively identify, remember, and compare sequences using LSR. LSR lacks readability and exhibits significant limitations in these aspects [13,14]. How to transform massive biological data into knowledge and how to update existing knowledge structures for efficient information retrieval have become new challenges [15,16]. Therefore, the development of sequence visualization and analysis systems that align with the characteristics of the current era has emerged as a new necessity. DNA sequence visualization (DNA SV) has opened up new avenues for biologists to extract information from massive datasets. At the same time, it has opened up a new research area in DNA sequence research [17,18]. DNA SV performs the local or global representation of sequences, extracting characteristic information from the sequences to enable multiple types of sequence analysis, such as sequence homology and similarity analysis [19,20]. As DNA SV brings convenience to various fields of biology, scientists have also extended visualization techniques to RNA and protein sequences. Therefore, research on DNA SV remains an important topic with profound implications for life sciences [21,22].

### 1.2. Focus and Vision of This Paper

**Figure 1:**
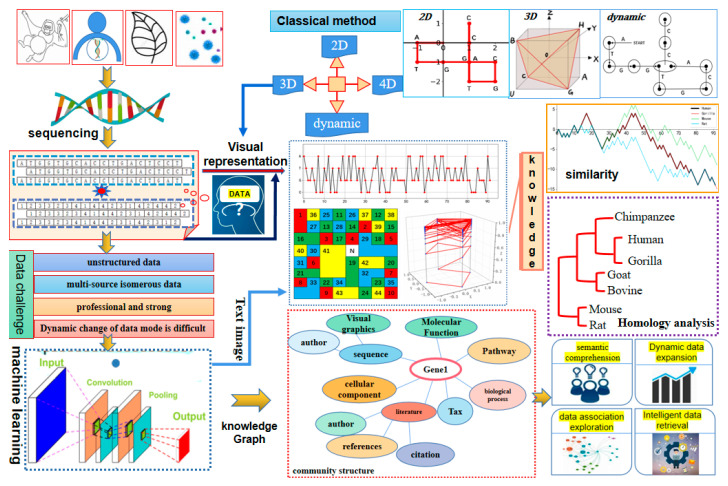
The overall framework of this review. This framework consists of two main parts: (1) Data visualization and knowledge extraction: This part involves 2D, 3D, 4D, and dynamic visualization methods to represent sequence characteristics. (2) Machine learning and knowledge integration: This part focuses on processing multi-source heterogeneous and unstructured data. Machine learning methods are used to extract features from these datasets. The extracted features are visualized to generate knowledge, which can further be organized into a knowledge graph.

This review primarily consists of two parts. The first part provides a comprehensive overview of DNA sequence visualization methods, focusing on two-dimensional, three-dimensional, high-dimensional, and dynamic visualization methods. Furthermore, the advantages and limitations of using visualization methods for sequence similarity measurement, gene mutation analysis, and other related analyses are discussed. The second part introduces two potential research directions: the construction of knowledge graphs for DNA sequence big data and the generation of DNA sequence graphics based on machine learning. This paper provides readers of DNA SV with a clear research path. The framework of this review is shown in Figure 1.

Traditional DNA sequence visualization methods are no longer efficient in extracting knowledge networks. There is an urgent need to find a method that can effectively explore the interconnected networks within massive data. A knowledge graph (KG) is an important branch of artificial intelligence that is used to represent concepts and their relationships in the physical world in a symbolic form [23,24,25]. Therefore, constructing a better KG system that organizes, manages, and understands the relationships between DNA sequences is of great importance for deep mining of massive data. Meanwhile, another challenge in DNA SV is the extraction of features from unstructured data [26]. Existing visualization methods for letter sequences suffer from subjective factors in feature extraction, as different methods may choose different features for visualization representation [27]. As a result, the generated graphics have different emphases and may not provide sufficient semantic information. Therefore, utilizing machine learning to construct visual representations of DNA sequences is another important research direction for the future.

## 2. Two-Dimensional (2D) Visualization

### 2.1. Random Walk Visualization

**Figure 2:**
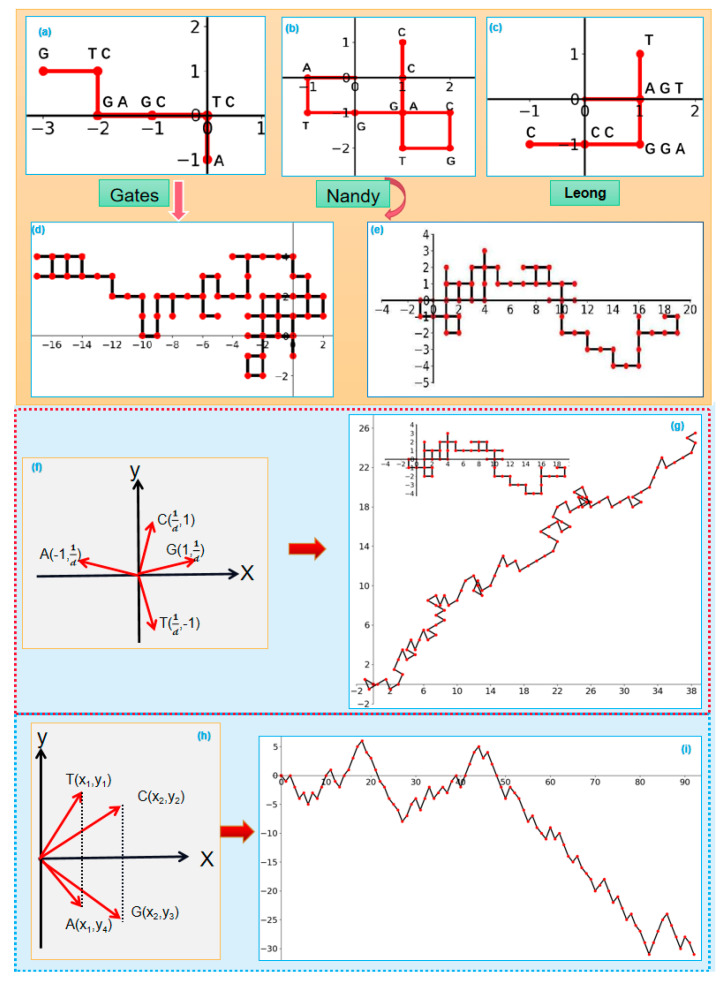
Walking-based visualization method. (**a**) The Gates graph of sequence fragment S. (**b**) The Gates–Nandy graph of sequence segment S. (**c**) The Leong and Morgenthaler graph of sequence segment S. (**d**) The Gates graph of sequence $\delta_{1}$. (**e**) The Gates–Nandy graph of sequence $\delta_{1}$. (**f**) The Guo theory model. (**g**) The Guo curve of sequence $\delta_{1}$. (**h**) The H-L curve theory model. (**i**) The H-L curve of sequence $\delta_{1}$.

A 2D visualization method for DNA sequences was proposed by Gates [28]. The basic principle is to assign the four basic nucleotides (adenine (A), thymine (T), guanine (G), and cytosine (C)) to a Cartesian coordinate system, constructing a symmetric purine–pyrimidine plot, as shown in Figure 2a. The six points in the figure correspond to the ten bases. The curves exhibit significant overlap, leading to pronounced information loss. For instance, points corresponding to repetitive bases tend to aggregate, making it challenging to distinguish sequence variations visually. To address these limitations, Nandy proposed a new 2D visualization method for DNA sequences, called the Gates–Nandy model [29,30]. In this model, Nandy introduced a different allocation scheme for the four nucleotides, as shown in Figure 2b. The curve structure in the figure is clear, and there is a noticeable improvement in reducing information loss. However, the appearance of closed loops introduces the issue of the number of cycles and the direction of traversal, which remains unknown [31]. To address the closed loop issue, Leong and Morgenthaler refined the nucleotide coordinate assignments (Figure 2c), reducing closed loops and improving curve interpretability. However, its complexity posed challenges for path traversal, and subsequent researchers have primarily focused on studying and improving the Gates and Gates–Nandy models. Figure 2d,e illustrate the practical performance of the Gates and Gates–Nandy models, respectively. Figure 2d demonstrates the clustering and repetitive paths present in the Gates model, particularly for long sequences, while Figure 2e highlights the improved clarity and reduced overlap achieved by the Gates–Nandy model. Figure 2e shows fewer closed loops and repetitive paths compared to Figure 2d. The Gates–Nandy model has an overall good visualization effect, but it still does not solve the problems of graph degeneracy and information loss in the visualization.

To address the information loss, many scholars have proposed improvement methods based on the Gates–Nandy model. Guo et al. used four special vectors in the Cartesian coordinate system to represent the four basic nucleotides, with each vector forming a fixed angle with the coordinate axes, as shown in Figure 2f. Figure 2g illustrates the visualization results of the improved algorithm for sequence visualization. The sequence curves constructed using the improved model exhibit an increasing pattern, with a reduced probability of self-crossing and overlapping, leading to a decrease in graph degeneracy. Especially, when compared to the Gates–Nandy graphical representation, the performance is significantly enhanced. However, Figure 2g is not strictly monotonically increasing, and the issue of degeneracy is not completely eliminated. Many improved methods based on the Gates–Nandy model have been proposed, such as the Leong and Morgenthaler models [32], as shown in Figure 2c. While they have improved the issue of degeneracy to some extent, they may also introduce new problems. The classical H-L curve representation allocates the four nucleotides into the first and fourth quadrants based on purines and pyrimidines [33], as shown in Figure 2h. The sequence curves constructed by this model completely solve the issues of overlap and crossing. The curves do not exhibit self-crossing or closed loops, eliminating the problems of degeneracy and information loss. At the same time, the number of projected points on the x-axis can reflect the length of the DNA sequence and also capture some detailed information within the sequence. However, in the case of long sequences, the sequence curves constructed by this model require a significant amount of space, which can still lead to visual clutter and loss of visual effectiveness, thus not fully achieving the desired goals.

### 2.2. Chaos Game Representation (CGR)

**Figure 3:**
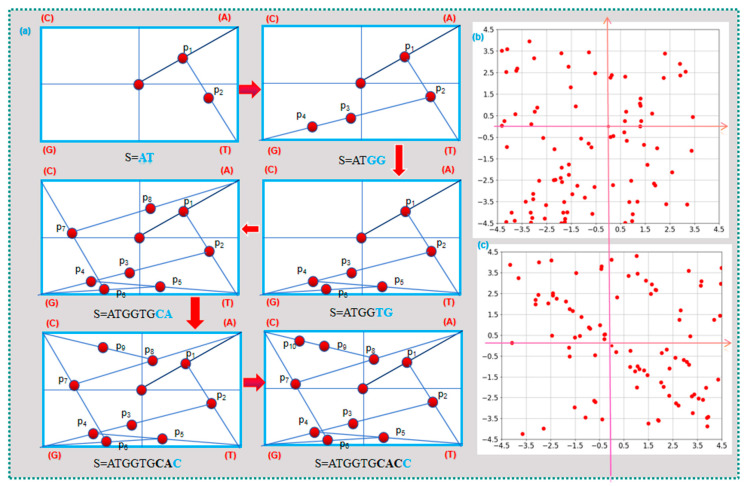
Chaos game representation visualization method. (**a**) The steps to construct a chaos game representation graph. (**b**) The chaos game representation graph of the sequence $\delta_{1}$. (**c**) The chaos game representation graph of the custom sequence φ.

The CGR visualization method abandons the traditional Cartesian coordinate system and instead adopts the principles of chaos theory. It uses scattered points within a square to represent the bases of a sequence [34]. This method has the advantage of being highly compact and occupying less space. Figure 3 illustrates the construction process of the visualization of CGR [35]. First, the four basic nucleotides are assigned to the four vertices of the square. Starting at the origin (0,0), the midpoint between the current point and the vertex corresponding to the current nucleotide is calculated and marked. This process is repeated iteratively for each nucleotide in the sequence, generating a fractal-like pattern. For example, in the sequence ”ATGG”, the first point p1 is the midpoint between (0,0) and the vertex for “A”. The second point p2 is the midpoint between p1 and the vertex for “T”. Similarly, p3 and p4 are calculated as the midpoints between the previous points (p2 and p3, respectively) and the vertex for “G”, as shown in Figure 3a. This method effectively visualizes nucleotide composition and sequence patterns [36]. The reverse construction process starts from the point corresponding to the last nucleotide in the sequence (denoted as p10). It involves moving in the reverse direction along the line connecting vertex A and point p10. The aim is to find the previous point, p9, that is equidistant from vertex A and p10. The nucleotide corresponding to p9 represents the second-to-last nucleotide in the sequence. By continuing this process, we can find the previous point one by one until all the points are identified, completing the reverse construction of the sequence. If the last point is unknown, you can choose any point within one of the quadrants as the starting point. The reverse construction process can be terminated when the point corresponding to the first base of the sequence is reached by reconstructing the local contour based on the starting point.

Figure 3b,c illustrate the visualization of CGR for different sequences. From Figure 3b, it can be observed that the points within the square appear to be random and without a specific pattern. If we divide the square into four quadrants following the principles of the Cartesian coordinate system, we can observe that the point corresponding to base A falls in the first quadrant, the point corresponding to base C falls in the second quadrant, the point corresponding to base G falls in the third quadrant, and the point corresponding to base T falls in the fourth quadrant. According to the density analysis of points in each quadrant, the content of the four basic nucleotides in the sequence can be determined. In Figure 3b, the density of points in the third quadrant is higher than in the other three quadrants, indicating a rich content of G nucleotides in the sequence. From Figure 3c, it can be inferred that the sequence has a higher content of C and T nucleotides, while the content of G and A nucleotides is significantly lower. Comparing Figure 3b,c, it can be observed that the two sequences have significant differences and a low similarity.

### 2.3. Double Vector and Double Nucleotide

**Figure 4:**
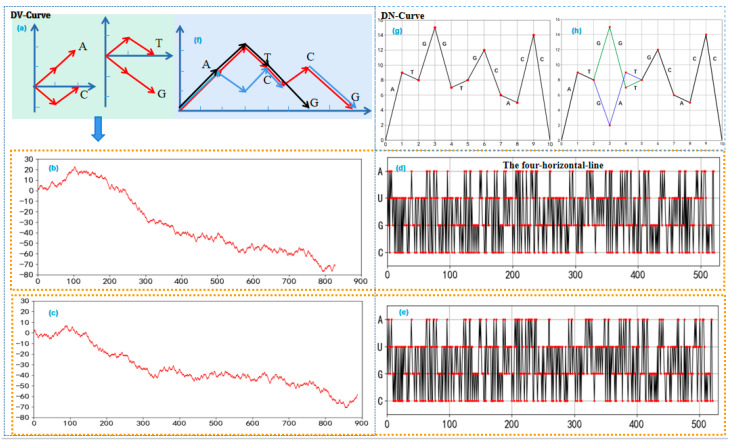
DV-Curve and DN-curve visualization methods. (**a**) Allocation of four nucleotides (A, T, C, and G) of DV-Curve in Cartesian coordinate system. (**b**) DV-Curve of sequence $\delta_{2}$. (**c**) DV-Curve of sequence $\delta_{7}$. (**d**) Four horizontal lines of sequence $\delta_{2}$. (**e**) Four horizontal lines of sequence $\delta_{7}$. (**f**) Genetic mutation DV-Curve analysis chart. (**g**) DN-Curve of sequence fragment S. (**h**) Comparison of DN-Curves for sequence segment S and sequence segment $L^{\prime }$.

Double Vector (DV-Curve) is a visualization method that uses two vectors to represent the basic nucleotides [37]. The method of constructing sequence graphs using the DV-Curve model is shown in Figure 4a. Figure 4a shows that the bases T and C do not move along the Y-axis in the coordinate system, while the bases A and G have displacements of two units up and two units down along the Y-axis, respectively. Therefore, the relationship between the quantities of G and A in a DNA sequence can be determined based on the positive, negative, or zero value of the curve at the Y-axis endpoint. To illustrate the advantages of using the DV-Curve for visualizing long sequences, we will compare the DV-Curve (as shown in Figure 4b) and the spectrum curve (as shown in Figure 4d) for a given sequence. Spectral visualization is one of the most popular two-dimensional methods, mainly consisting of the four-horizontal-line method and the two-horizontal-line method. By comparing the two figures, it can be observed that for sequences longer than 300 bp, the DV-Curve method maintains a good visual representation without any loss of information [38]. In contrast, the spectrum representation tends to lose its visual advantage as the sequence length increases. The DV-Curve and the spectrum curve for the sequence $\delta_{7}$ are shown in Figure 4c,e, respectively. It is observed that directly determining similarity through the comparison of the spectrum curve (Figure 4d,e) is quite challenging. However, from the DV-Curve (Figure 4b,c), it is apparent that there are significant differences between the DNA sequences. This indicates that, for long sequences, the DV-Curve has better visualization capabilities compared to the spectrum representation method.

Indeed, the DV-Curve method has significant applications in gene mutation analysis. It assesses the presence of gene mutations by plotting the original sequence and the potential gene mutation sequence on the same Cartesian coordinate system and observing whether the DV-Curves of the two sequences overlap, as shown in Figure 4f. By observing the changes in the length of the DV-Curve, it is possible to infer the type and location of gene mutations. If the length of the DV-Curve increases, it indicates an insertion mutation where additional bases have been inserted into the sequence. If the length decreases, it suggests a deletion mutation where some bases have been removed from the sequence. If the length remains unchanged but the positions do not overlap, it signifies a substitution mutation where one or more bases have been replaced with different bases.

Double Nucleotide (DN-curve) visualization is a method of visualizing sequences using all 16 possible combinations of base pairs [39]. Through this visualization method, we are able to observe the relative positions and spatial relationships between base combinations, which provides unique advantages for understanding the structure and patterns of the sequence as well as identifying important features within the sequence [40,41]. The DN-curve holds clinical significance for the study, diagnosis, and treatment of genetic diseases. For instance, in mutation detection, the DN-curve identifies mutations by comparing the differences between an individual’s genomic sequence and a reference genome sequence. On the curve, each point represents the position of a nucleotide, and the height of the curve denotes the frequency of a specific base observed at that location. The mathematical model of the sequence is given as follows:

(1) $$\varphi (s_{i}s_{i+1})=\left\{\begin{array}{c} (i,1)\;\mathrm{if}\;\;s_{i}s_{i+1}=\mathrm{AG}\\ (i,2)\;\mathrm{if}\;\;s_{i}s_{i+1}=\mathrm{GA}\\ (i,3)\;\mathrm{if}\;\;s_{i}s_{i+1}=\mathrm{CT}\\ (i,4)\;\mathrm{if}\;\;s_{i}s_{i+1}=\mathrm{TC}\\ (i,5)\;\mathrm{if}\;\;s_{i}s_{i+1}=\mathrm{AC}\\ (i,6)\;\mathrm{if}\;\;s_{i}s_{i+1}=\mathrm{CA}\\ (i,7)\;\mathrm{if}\;\;s_{i}s_{i+1}=\mathrm{GT}\\ (i,8)\;\mathrm{if}\;\;s_{i}s_{i+1}=\mathrm{TG}\\ (i,9)\;\mathrm{if}\;\;s_{i}s_{i+1}=\mathrm{AT}\\ (i,10)\;\mathrm{if}\;\;s_{i}s_{i+1}=\mathrm{TA}\\ (i,11)\;\mathrm{if}\;\;s_{i}s_{i+1}=\mathrm{CG}\\ (i,12)\;\mathrm{if}\;\;s_{i}s_{i+1}=\mathrm{GC}\\ (i,13)\;\mathrm{if}\;\;s_{i}s_{i+1}=\mathrm{AA}\\ (i,14)\;\mathrm{if}\;\;s_{i}s_{i+1}=\mathrm{CC}\\ (i,15)\;\mathrm{if}\;\;s_{i}s_{i+1}=\mathrm{GG}\\ (i,16)\;\mathrm{if}\;\;s_{i}s_{i+1}=\mathrm{TT} \end{array}\right.$$

where i represents the position of the base in the sequence; i=1,2,3⋯N−1. In a Cartesian coordinate system, the coordinates of the base pair combinations are marked on the Cartesian coordinate system, and adjacent points are connected to form a DN-Curve, as shown in Figure 4g. The DN-Curve can highlight the features of base pair combinations, which is highly advantageous for studying the distribution of mutations in base pair combinations. Figure 4h depicts the DN-Curve of two sequences in the same coordinate system. The coordinate of the base pair combination s3s4 in the sequence S is (3,15), while the coordinate of the base pair combination s3s4 in the sequence $S^{\prime }$ is (3,2). The vertical coordinate difference is s4 = 15 − 2 = 13. Therefore, it can be inferred that there is a mutation in the fourth base of the sequence.

### 2.4. Spectral Visualization

**Figure 5:**
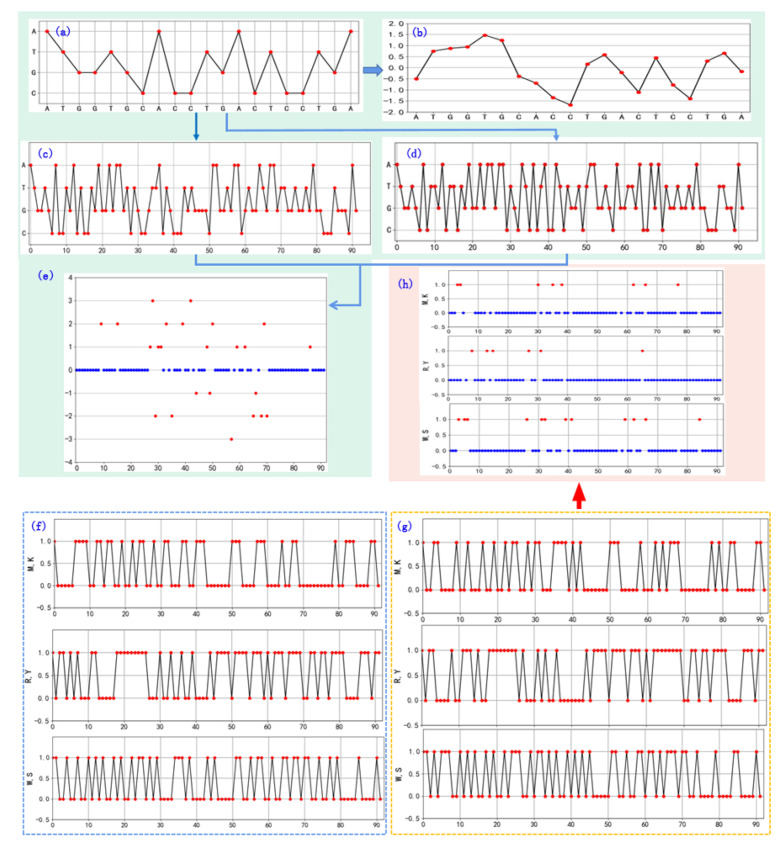
Spectral visualization method. (**a**) Four horizontal lines of sequence fragment γ. (**b**) Chaotic four horizontal lines of sequence fragment γ. (**c**) Four horizontal lines of sequence $\delta_{1}$. (**d**) Four horizontal lines of sequence $\delta_{7}$. (**e**) Differences between $\delta_{1}$ and $\delta_{7}$. (**f**) Two horizontal lines of sequence $\delta_{1}$. (**g**) Two horizontal lines of sequence $\delta_{3}$. (**h**) Differences between $\delta_{1}$ and $\delta_{3}$.

Spectral visualization is one of the most popular two-dimensional methods, mainly consisting of the four-horizontal-line method and the two-horizontal-line method. The four-horizontal-line method does not require pre-assigning four basic nucleotide vectors in the Cartesian coordinate system [42]. First, four horizontal lines are drawn at equal distances, and then these four lines are labeled according to the order in which the four nucleotides appear in the sequence, as shown in Figure 5a. The advantage of this method is that it can visually demonstrate the distribution of bases in the sequence, avoiding the degeneracy and degeneration issues of traditional visualization methods in the Cartesian coordinate system. Additionally, this method can also reflect the local features and similarities of the sequence. Figure 5b illustrates a variant of the four-horizontal-line method, known as the chaotic four-horizontal-line method [43,44]. This method integrates chaos theory to calculate the position of each nucleotide and map it onto a coordinate system. The position of each nucleotide is determined by the coordinate of the preceding nucleotide and its corresponding chaotic factor. Compared to the traditional four-horizontal-line method, the chaotic four-horizontal-line method effectively avoids overlapping points on the vertical axis, providing a clearer representation of local sequence features. This visualization approach intuitively captures local patterns in sequences, such as nucleotide clustering or repetitive structures, supporting biological function analysis and sequence similarity studies.

The four-horizontal-line visualization method can be employed to analyze the similarity of sequences. Figure 5c,d illustrate the construction of four-horizontal-line representations for two DNA sequences. By comparing the two curves, it can be observed that there are significant nucleotide differences between the intervals 28–71. Figure 5e depicts the difference plot between two sequences, where the points on the X-axis (y=0) in the figure represent matching bases between the two sequences, and the skipped points (y≠0) represent non-matching bases between the two sequences.

The two-horizontal-line method is a visual representation method based on the chemical properties of nucleotides, where three sets of two horizontal lines are used to construct graphical curves as a visual representation [45,46]. Nucleotides are classified into three categories (purine R = [A,G]/pyrimidine Y = [C,T], amino M = [A,C]/keto K = [ G,T], and weak-H bond W = [A,T] /strong-H band S = [C,G]). The two-horizontal-line method allows for the observation and analysis of the chemical and structural features of a sequence through a simple and intuitive graphical representation. Figure 5f,g depict the graphical representations of different sequences using the two-horizontal-line method. Figure 5h illustrates the difference plot of two sequences. In Figure 5h, the W/S curve shows the difference in the number of bases with strong and weak hydrogen bonds between the sequences. The M/K curve displays the difference in the positions of amino and keto groups. Additionally, the R/Y curve shows the difference in the positions of purine and pyrimidine bases between the sequences. Therefore, the three sets of feature curves can serve as a coarse-grained description of the DNA primary sequence and can be used for sequence analysis and reconstruction. The visualization effect of this method is intuitive, as it can clearly display the distribution of different types of bases in the sequence and provide a deeper understanding of the structure and chemical properties of DNA sequences. Additionally, this method converts sequences into numerical features, which requires relatively less computational resources. This allows computers to process and compare the sequences more quickly [47].

### 2.5. Worm Curves and Five-Color Map

**Figure 6:**
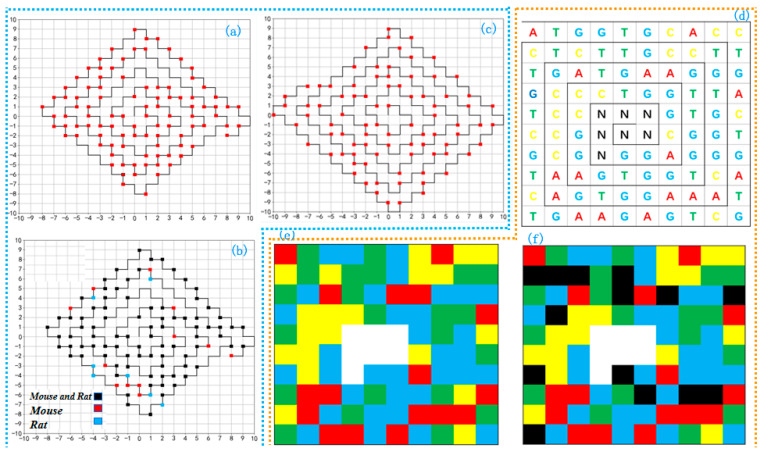
Worm curves and five-color map visualization method. (**a**) WormBin curve of sequence $\delta_{4}$. (**b**) Differences between $\delta_{4}$ and $\delta_{5}$. (**c**) WormStep curve of sequence $\delta_{6}$. (**d**) Square curve of sequence $\delta_{5}$. (**e**) Five-color map of sequence $\delta_{5}$. (**f**) Differences between $\delta_{4}$ and $\delta_{5}$.

The worm curve visualization method (WormBin) represents a sequence of length N in a $\sqrt{n}*\sqrt{n}$ space [48]. The resulting graphical representation is relatively compact, allowing for the display of longer DNA sequences within a smaller space. It provides an intuitive visualization of the positions and relative relationships of different bases [49]. First, the bases A, G, C, and T are encoded as binary labels 00, 01, 10, and 11, respectively, and mapped onto consecutive vertices of a predefined worm curve. This 2D path follows a consistent rule: turning 90° right if unvisited, or 270° left otherwise, ensuring no self-intersections. The path is identical for all sequences and arranged compactly on a 2D plane without biological significance for vertex positions. Only ‘1’ bits are marked as red dots, with multiple ‘1’ bits represented independently. This results of WormBin representation are shown in Figure 6a. Each base in the WormBin has two adjacent vertices, and the curve does not intersect itself, avoiding degeneracy and ambiguity. Figure 6b depicts the worm curves of two sequences in the same Cartesian coordinate system. Black dots represent identical bases in both sequences, while blue and red dots represent different bases in the two sequences. By counting the number of different points, the similarity between two sequences can be directly calculated. The WormBin method can visually demonstrate the similarity and differences between sequences without requiring any special preprocessing of the sequences.

The WormBin method has a notable limitation: it is not able to quickly identify which base corresponds to a specific point on the curve, making it difficult to reconstruct the DNA sequence or perform local analysis. To address this issue, the WormStep visualization method for DNA sequences has been proposed [50]. The construction method of WormStep is overall similar to the rules of the WormBin curve, as shown in Figure 6c. In this process, the four basic nucleotides are labeled on the curve based on the number of steps taken. If it is necessary to determine the corresponding nucleotide represented by a point in the graph, it can be identified by the number of steps between it and the previous point. WormStep overcomes the drawback of WormBin in not being able to quickly identify the bases, making sequence reconstruction and local sequence analysis easier.

The WormBin and WormStep visualization methods are effective for short DNA sequences, but they are not suitable for extremely long sequences. To overcome this limitation, the four-color map and five-color map visualization methods have been proposed (five-color map is an improvement of the four-color map). In the case of the five-color map, as shown in Figure 6d, a regular square curve is generated, and the starting position of the bases begins from the top left corner of the square curve, and the bases are allocated to small squares in a clockwise direction. The four nucleotides and the central blank space are marked with different colors. This method provides a better visualization effect. To better observe local features, adjacent small squares with the same base are merged, and then the merged regions are colored according to the color rules (blue for G, yellow for C, green for T, red for A, and white for N), as shown in Figure 6e. The colored graph clearly reveals the distribution and quantity of each base, with a higher abundance of G bases. At the same time, each small square corresponds to a base in the sequence, and there is no degeneracy issue. Additionally, the color-coded representation allows people to easily identify the differences between two images, making it a valuable tool for genetic mutation analysis and similarity analysis. Using this method to analyze the similarity of two sequences, as shown in Figure 6f, it is easy to identify the differing bases. The differing bases are marked in black. The similarity analysis results of the two sequences are consistent with other methods.

## 3. Three-Dimensional (3D) Visualization

### 3.1. Z-Curve

**Figure 7:**
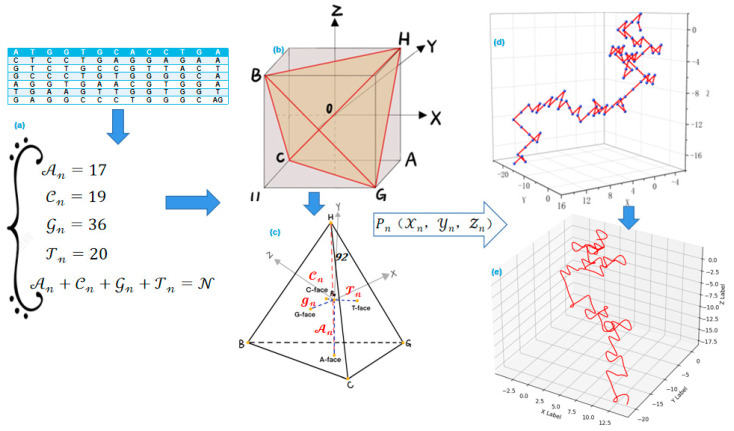
Z-curve construction framework for sequence $\delta_{1}$. (**a**) The nucleotide counts (A, C, G, T) are computed cumulatively along the sequence. (**b**) The tetrahedral framework maps nucleotide composition into a three-dimensional coordinate system. (**c**) The 2D projection of the tetrahedral framework demonstrates the contributions of each nucleotide to the overall sequence structure. (**d**) The initial Z-curve is constructed as a three-dimensional trajectory based on cumulative nucleotide counts. (**e**) The smoothed Z-curve is obtained by applying B-spline functions to the trajectory of the initial Z-curve.

The Z-curve is a classical method that converts DNA sequences into a visually intuitive geometric shape, forming a unique folded three-dimensional curve representation [51]. The Z-curve method overcomes some limitations of traditional algebraic methods and exhibits strong uniqueness and reversibility. It is advantageous for the visualization and analysis of sequences, allowing for a visual observation of the structure and features of DNA sequences, such as base pairing, sequence folding, and circular structures, among others [52,53]. The construction process of the Z-curve is depicted in Figure 7.

Let $A_{n}$, $T_{n}$, $G_{n}$, and $C_{n}$ represent the cumulative counts of bases A, C, G, and T, respectively, up to position n in a DNA sequence of length N. In a three-dimensional coordinate system, we can find a point $P_{n}$ that uniquely represents a base in a DNA sequence. To facilitate the calculation of the coordinates of points $P_{n}$ in space, we establish a regular tetrahedron BCGH inside the cube with a height equal to L, as shown in Figure 7b. Each face of the tetrahedron corresponds to a nucleotide (A, T, G, C), and the sum of distances from any point to the four faces equals the tetrahedron’s height. The 2D projection of the tetrahedral framework demonstrates the contributions of each nucleotide to the overall sequence structure, as shown in Figure 7c. The coordinates of point $P_{n}$ within the regular tetrahedron can be represented as ($X_{n},Y_{n},Z_{n}$). The coordinate calculation formula for point $P_{n}$ within the regular tetrahedron is as follows [54]:

(2) $$\left\{\begin{array}{c} Xn=(A_{n}+G_{n})-(C_{n}+T_{n})\\ Yn=(A_{n}+C_{n})-(G_{n}+T_{n}),\\ Zn=(A_{n}+T_{n})-(C_{n}+G_{n}) \end{array}\right.\;n=0,1,2,3,\cdots ,N$$

In Formula (2), the coordinates of point $P_{n}$ are calculated based on the cumulative counts of nucleotides up to position n in the sequence. These cumulative counts reflect the nucleotide composition from the sequence start to position n. Next, mark these coordinates in 3D space and connect them with lines between adjacent points. This will result in the Z-curve representation of the DNA sequence. The directly constructed Z-curve may not be smooth. To achieve a smooth representation, the B-spline interpolation method can be applied. For example, Figure 7d shows the initial Z-curve of sequence $\delta_{1}$, constructed based on cumulative nucleotide counts. Figure 7e presents the smoothed version of this curve after applying B-spline interpolation, improving the clarity of local and global structural features.

The Z-curve reveals rich spatial folding structures that reflect nucleotide distribution characteristics, including symmetry, periodicity, and local/global features. These features are particularly useful for analyzing repetitive DNA sequences and comparing sequence patterns. By examining the folding and periodicity of Z-curves from different sequences, researchers can identify similarities, differences, and structural variations [55].

### 3.2. L-Curve

**Figure 8:**
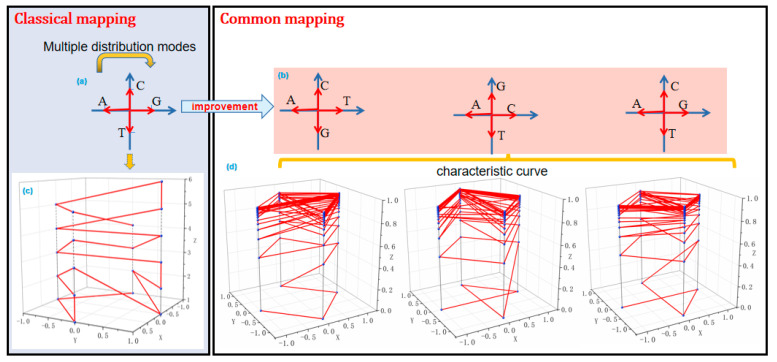
L-curve visualization method. (**a**) Allocation of four basic nucleotides in two-dimensional coordinate system. (**b**) Four nucleotide vector assignment patterns for improved L-curve method. (**c**) Normal L-curve of sequence fragment γ based on initial nucleotide allocation modes. (**d**) Improved L-curve of sequence $\delta_{6}$ based on optimized nucleotide allocation modes.

The L-curve method has a low computational cost and does not require three-dimensional projection. It can effectively display the structure and features of DNA sequences in a clear manner. The construction process of the L-curve is simple [56,57]. Firstly, it involves mapping the four basic nucleotides onto a two-dimensional coordinate system to determine their plane coordinates, as shown in Figure 8a. The arrow labeled “multiple distribution modes” indicates that the four nucleotides (A, C, G, and T) can be mapped to different positions in the coordinate system using various allocation schemes. Among these, three commonly used allocation schemes are demonstrated in Figure 8b, each representing a distinct characteristic representation of the nucleotide distribution. Then, iterate through the DNA sequence, and, for each nucleotide, calculate its cumulative occurrence count, which serves as the coordinate value along the Z-axis. Finally, by connecting the adjacent points, the three-dimensional curve graph representing the entire sequence is obtained based on different nucleotide mapping schemes, as shown in Figure 8c,d. The graph clearly shows the positions and quantities of each nucleotide, with a simple structure, and the reconstruction of the DNA sequence is relatively straightforward.

A potential issue with the L-curve method is that there are multiple ways to allocate the planar coordinates for the four basic nucleotides, while different allocation schemes may have an impact on the resulting L-curve [58]. Researchers have proposed an improved L-curve algorithm based on the classification of nucleotides according to their chemical properties. This enhancement aims to provide a more accurate representation of the structure and features of DNA sequences in the L-curve. The improved L-curve algorithm utilizes three different vector representation modes to represent the four basic nucleotides, as shown in Figure 8b. Each DNA sequence corresponds to three L-curve modes, thereby resolving the issue of non-uniqueness in the allocation of plane coordinates in the L-curve method. Figure 8d depicts the L-curve curve with the three modes included. By calculating the geometric center of the L-curve curves for the three modes, a 9D vector can be constructed for sequence similarity measurement [30,59]. If two vectors in the 9D space point in similar directions, then the two DNA sequences are considered similar. This sequence comparison method is simple to calculate and significantly reduces computational complexity.

### 3.3. DN-Curve 3D

**Figure 9:**
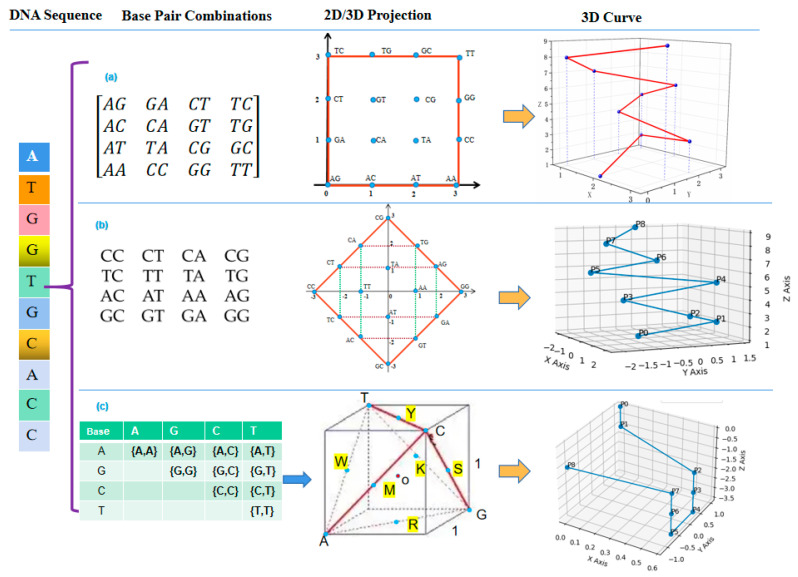
DN-Curve 3D visualization method. (**a**) Chemical property-based visualization principle of 16 base combinations divided into four classes. (**b**) Molecular weight-based visualization principle of 16 base combinations divided into three classes. (**c**) Ten-base combination visualization principle.The process of using the 10-base pair combination DN-Curve 3D method can be described as follows: In this method, the 10 base pair combinations are represented as points mapped onto a regular tetrahedron in three-dimensional space. These points correspond to the vertices and midpoints of the edges of the tetrahedron, forming 10 fixed positions. Each point defines a unique vector originating from the centroid of the tetrahedron $\left(O\left(0,0,0\right)\right)$ and terminating at the corresponding point on the unit sphere. These vectors are normalized to ensure they are unit vectors, meaning all vectors have a length of 1. The z-coordinate for each point in the DN-Curve 3D method is determined by the positional index of the corresponding base pair within the DNA sequence. By connecting the points in three-dimensional space, a curve is constructed to represent the DNA sequence, as depicted in Figure 9c. The spatial arrangement of the points and their associated vectors provides a clear mapping for each base pair combination. This facilitates the visual observation and analysis of the DNA sequence features, including the spatial distribution and structural characteristics of base pair combinations within the DNA sequence.

The Double Nucleotide Curve (DN-Curve 3D) visualization method combines consecutive pairs of nucleotides in the DNA sequence as a single unit and maps it to a point in three-dimensional space [60,61]. By connecting all the points, a curve is formed. The DN-Curve 3D method has 16 possible combinations for DNA sequences consisting of four nucleotides (A, C, G, T) and 10 possible combinations for DNA sequences. The 16 possible combinations of base pairs in the DN-Curve 3D method can be categorized using two different classification methods. (1) Chemical property-based classification (16 combinations): This method categorizes the 16 possible combinations of base pairs into four classes based on the chemical properties of the bases, as shown in Figure 9a. A 4 × 4 matrix is then constructed to represent these categories. Next, the three-dimensional curve is constructed by determining the planar coordinates for each base pair combination within the two-dimensional coordinate system [62]. (2) Molecular weight-based classification (16 combinations): This method categorizes the 16 base pair combinations into three groups based on the molecular weights of the four nucleotides, as shown in Figure 9b. Similarly, a 4 × 4 matrix is constructed to represent these categorizations. In this method, the 16 base pair combinations are divided into three groups along the X-axis in the coordinate system. The coordinates of each base pair are determined based on this division, and a three-dimensional curve is constructed using these coordinates [63]. The z-coordinate in the DN-Curve 3D graph is determined by the positional index of the base pair within the DNA sequence.

### 3.4. C-Curve

**Figure 10:**
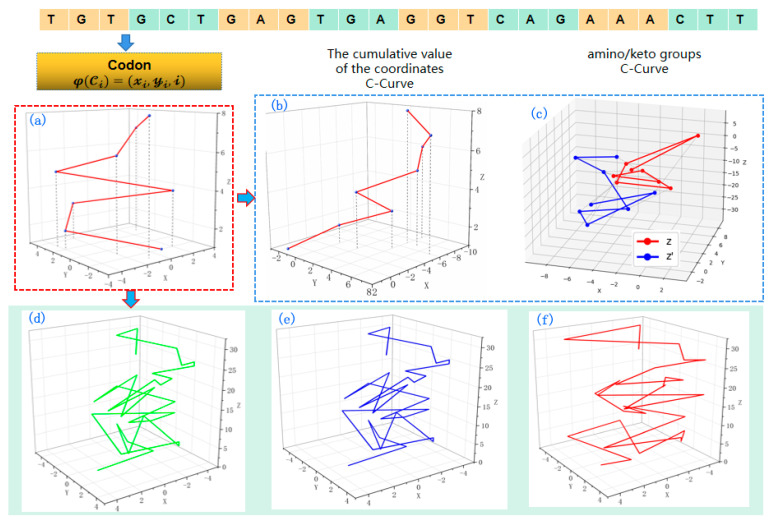
C-Curve visualization method. (**a**) C-Curve of sequence fragment H. (**b**) Cumulative C-Curve of H. (**c**) Variant C-Curve of H. (**d**) C-Curve of sequence $\delta_{1}$. (**e**) C-Curve of sequence $\delta_{2}$. (**f**) C-Curve of sequence $\delta_{3}$.

The C-Curve technique is a visualization method that maps codons to three-dimensional space. C-Curve is used to display the distribution of codons in DNA sequences and extract certain descriptors that represent both global and local information of the DNA sequence [64]. It assists researchers in studying the encoding information of genomes. Firstly, C-Curve converts the DNA sequence into codon form. Then, each codon is transformed into a set of points, which are mapped to a three-dimensional coordinate system [65]. In the three-dimensional coordinate system, the points are labeled based on their coordinate values, and the adjacent points are connected to form the C-Curve, as shown in Figure 10a. The coordinate relationships for the nucleotides in a given codon are as follows: For the first nucleotide, if x>0, it is either A or T; otherwise, it is C or G. If y>0, it is either A or G; otherwise, it is C or T. If x>0 and y>0, it is A. For the second nucleotide, its identity is determined by the projection onto the xz-plane (the sign of x and z), while the third nucleotide is determined by the projection onto the yz-plane (the sign of y and z). The z-coordinate corresponds to the codon’s position in the sequence and increases linearly, ensuring that the sequential order of codons is preserved in the 3D representation.

The alternative invariants inferred by C-Curve can generate various other forms of curves, providing multiple effective methods for sequence information mining. For example, by calculating the cumulative coordinate values of codons, a cumulative effect plot of codons can be generated, as shown in Figure 10b. By referring to Figure 10b, we can gain insights into the global information of the sequence and infer that there is a higher abundance of codons starting with the nucleotides G or C. Additionally, we can also plot z = x*y and the cumulative value of z to distinguish amino/keto groups, as shown in Figure 10c.

The C-Curve method has significant advantages in the application of sequence similarity measurement [66]. Figure 10d–f depict the C-Curves of different sequences. The curves show that sequence $\delta_{1}$ (the first exon of human β-globin gene) and sequence $\delta_{2}$ (the first exon of Gorilla β-globin gene) are similar. The problem with C-Curve is that when describing very long DNA sequences, it can lose some visual advantages, and comparing them can be complex and time-consuming.

## 4. Dynamic Visualization

“Dynamic” refers to the ability of visualization methods to dynamically adjust based on the structure, sequential complexity, and characteristics of DNA sequences. Such dynamic methods are particularly useful for sequence similarity analysis, gene function annotation, classification and clustering, as well as applications in drug design and biomedical research. For instance, key features such as periodic patterns, frequency distributions, information entropy, and dynamic changes can be extracted from the spectrum. These features not only comprehensively reflect the global structure and local details of the sequence but also significantly reduce redundant information. Moreover, these features demonstrate a distinct advantage in addressing scalability issues. By extracting high-information-density dynamic features, the original base sequences can be transformed into low-dimensional matrices, thereby reducing the scale of input data and decreasing the computational burden on models. At the same time, these dynamic features are highly compatible with distributed computing frameworks.

### 4.1. Spectral Dynamic Visualization

**Figure 11:**
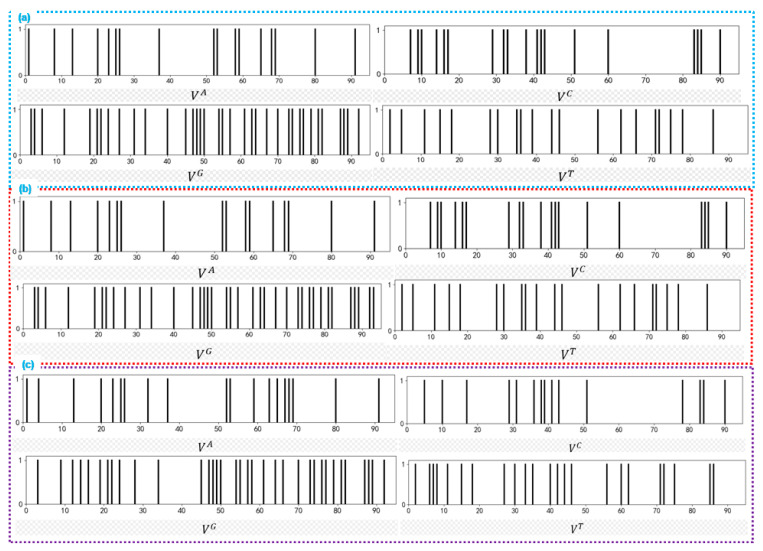
The spectral dynamic method. (**a**) The B-spectrum of sequence $\delta_{1}$. (**b**) The B-spectrum of sequence $\delta_{2}$. (**c**) The B-spectrum of sequence $\delta_{3}$.

Spectral dynamic visualization incorporates spectroscopy and kinetic theory into DNA sequence analysis. It represents the four basic nucleotides using a set of discrete line segments, constructing four sets of B-spectra [67]. Each nucleotide corresponds to a discrete line segment in the B-spectrum. Figure 11a–c depict the spectral dynamic representations of different sequences. The positions of the line segments in the figures represent the positions of the bases in the DNA sequence [68]. The density and positions of the line segments in the B-spectrum provide information about the distribution of specific bases in the DNA sequence [69]. From the B-spectrum, it can be observed that the three sequences have a significantly higher content of the base G. The content of G + C is noticeably higher than A + T in all three sequences. Therefore, it can be inferred that the first exon sequences of the β-globin gene in these three species have a high GC content.

Spectral dynamic visualization has the advantage of being simple and easy to use in sequence quantification analysis. By calculating the rotational inertia of the four sets of B-spectra, four descriptors of the sequence can be obtained [70,71]. These four descriptors can be combined to form a 4D vector representing the sequence. By calculating the similarity index of the 4D vector, we can quantitatively compare the similarity between sequences and quantify the similarity between sequences. Indeed, this method can be applied in various fields, including bioinformatics and chemistry. For example, it is used to compare the similarity of DNA sequences, protein sequences, or chemical structures. The computational complexity of the spectral dynamic graph is not affected by the length of the sequence, making it suitable for addressing similarity measurement problems in long sequences. So, it has significant advantages in addressing large-scale DNA sequence alignment problems.

### 4.2. Two-Dimensional Dynamic Visualization

**Figure 12:**
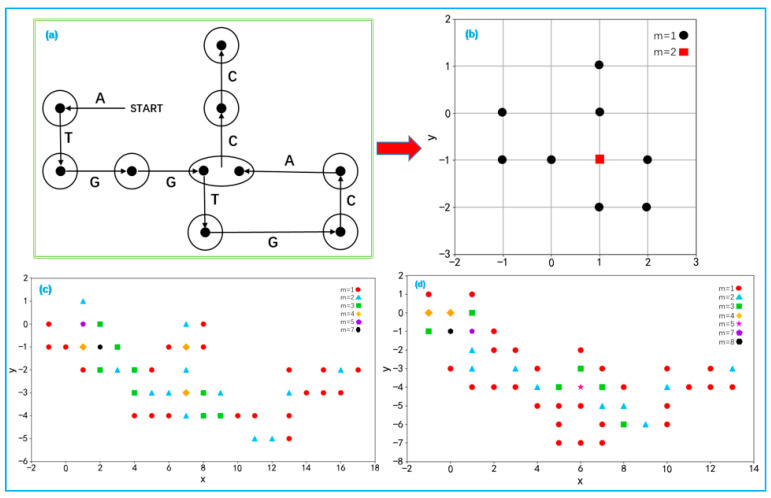
Two-dimensional dynamic visualization method. (**a**) Process of constructing a 2D dynamic visualization of sequence fragment S. (**b**) Two-dimensional dynamic graph of sequence S. (**c**) Two-dimensional dynamic graph of sequence $\delta_{4}$. (**d**) Two-dimensional dynamic graph of sequence $\delta_{5}$.

Two-dimensional dynamic visualization represents a sequence using points of different qualities. This approach effectively addresses the issue of degeneracy in sequences. By using points of different qualities, the traversal path of each base in the coordinate system can be clearly visualized, making the structure and features of the sequence more distinct and visible [72]. Unlike traditional static methods, the dynamic approach represents sequences as sets of material points with different qualities, such as mass, to capture more nuanced details of the sequence’s structural and spatial properties. The construction process involves three main steps: First, construct the random walk graph for the bases, as shown in Figure 12a. Secondly, determine the quality of the points, where each ellipse represents the endpoint of a vector, and the number of points inside the ellipse represents the quality of that point. Finally, construct a dynamic graph based on the quality of each point, as shown in Figure 12b. This graph clearly visualizes traversal paths and base positions, achieving a two-dimensional dynamic representation of the DNA sequence.

This representation method has found applications in the field of DNA sequence analysis. Two-dimensional dynamic visualization can visually display the similarity and diversity between species, revealing the evolutionary relationships and history among species and constructing phylogenetic trees to study the temporal evolution of species [73,74]. Figure 12c,d present the two-dimensional dynamic representations of different sequences, providing a framework to compare their structural characteristics. The similarity between these sequences can be observed by analyzing specific patterns in the graphs. For instance, the clustering patterns of points (distinguished by color and shape based on their masses) reveal the distribution characteristics of the sequences. Additionally, the overall shapes, including the trajectories and spreads of points in the x−y plane, highlight structural similarities. While minor variations in clustering or distribution may exist, the consistent trends in point clustering and spatial distributions suggest a high degree of visual similarity between the two sequences. However, for longer DNA sequences, the visualizations may become complex and difficult to directly compare. In such cases, it is necessary to extract sequence descriptors from the two-dimensional dynamic visualizations. Centroid and moment of inertia serve as sequence descriptors that quantitatively characterize the spatial distribution of nucleotides in the 2D dynamic graph. The centroid represents the weighted average position of all nucleotides in the sequence, reflecting the overall nucleotide composition and bias. The moment of inertia measures the spread of nucleotides relative to the centroid, capturing structural features such as clustering, symmetry, and distribution patterns. These descriptors enable more precise sequence comparisons by providing numerical values that reflect both global and local structural characteristics of the sequences.

### 4.3. Coded Mark Inversion (CMI) Encoding Dynamic Visualization

**Figure 13:**
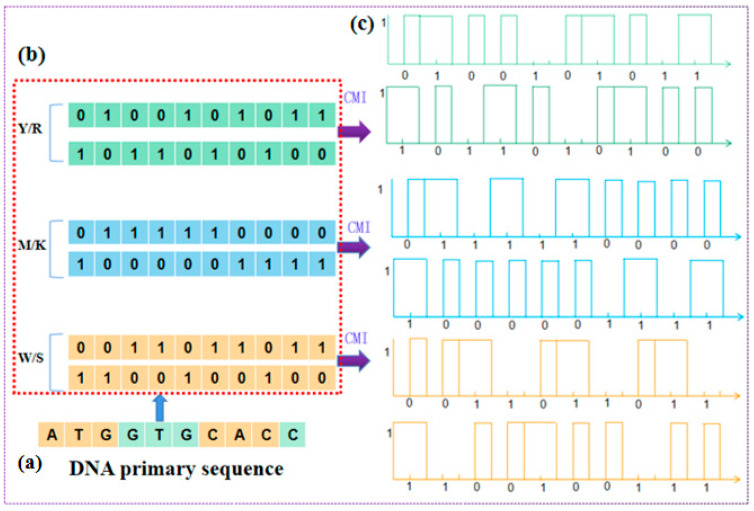
A principle diagram of the CMI encoding visualization method. (**a**) The original DNA sequence is displayed at the bottom. (**b**) Three binary encodings are generated based on the nucleotide classifications: Y/R (pyrimidine/purine), M/K (amino/keto), and W/S (weak/strong hydrogen bonding). (**c**) The CMI representation.

The CMI encoding dynamic visualization method categorizes DNA sequences based on the chemical properties of the bases [31]. It translates DNA sequences into source sequences and converts the source sequences into a 0–1 encoded sequence. On the contrary, the source sequences are obtained by reverse-translating the 0–1 encoded sequence. Based on chemical properties, nucleotides can be divided into three groups, with each group containing two types [75,76]. Therefore, we can obtain six different sets of CMI-encoded graphics, with each set corresponding to a specific combination of bases, as shown in Figure 13.

CMI encoding dynamic visualization integrates both the chemical properties and structural features of DNA sequences. Not only does it provide an intuitive way to observe and analyze the features of DNA sequences, but it also allows for the extraction of structural and chemical information from the sequences [77]. Additionally, this representation method allows for quantitative measurement of sequence similarity with relatively low computational cost, providing valuable clues for subsequent sequence analysis and functional research.

## 5. Four-Dimensional Numerical Representation

### 5.1. The Bandwidth Numerical Representation

The numerical representation of bandwidth is a method of representing sequences using the bandwidth invariant. By calculating the average of the off-diagonal elements in the distance matrix, a set of numerical features called “band invariants” is obtained [78]. These band invariants can be used for comparing and analyzing sequences. The numerical representation method of bandwidth is simple and efficient, allowing for the fast analysis and processing of DNA sequences [79]. To construct a distance matrix, the Euclidean distance between two bases in a sequence fragment S is calculated as follows:

(3) $$\sqrt{\left\{({A_{i}-A_{j})}^{2}+({T_{i}-T_{j})}^{2}+({G_{i}-G_{j})}^{2}+({C_{i}-C_{j})}^{2}\right\}}$$

Here, $A_{i}{,\;T}_{i},\;G_{i}\;and\;C_{i}$ represent the coordinate values of the i-th base in the sequence fragment S, where i=1,2,3,⋯n. Similarly, $A_{j}{,\;\;T}_{j},\;\;G_{j}\;and\;C_{j}$ represent the coordinate values of the j-th base in the sequence fragment S, where j=1,2,3,⋯n.

**Figure 14:**
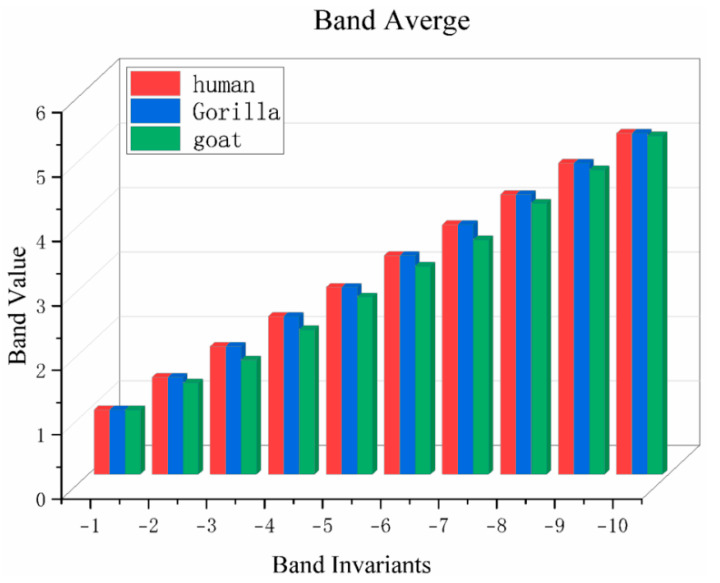
Bar chart of average bandwidth for a 10 × 10 fragment distance matrix of sequences $S,S^{\prime }$, and $S^{\prime \prime }$.

To quantitatively calculate the similarity of DNA sequences, invariant features are extracted from the distance matrix. Figure 14 shows the bar plot of the average bandwidth values from the distance matrices of three sequence fragments [80,81]. Figure 14 clearly demonstrates that the average bandwidth values of sequence fragments $S\;and\;S^{\prime }$ in the distance matrices are very similar, while they exhibit significant differences compared to sequence fragment $S^{\prime \prime }$. It can be noted that the bandwidth invariants of different DNA sequences exhibit considerable variations in both their form and numerical values. In any local feature analysis, it is possible to set different bandwidths for calculation [31,42]. However, using different values may result in variations in local similarity.

In Figure 14, the horizontal axis (band invariants) represents the band invariants, which correspond to the indices of different off-diagonals in the distance matrix. For example, −1 represents the first off-diagonal of the distance matrix, −2 represents the second off-diagonal, and so on. Each diagonal represents the distances between different base pairs, with smaller indices indicating larger distance spans between the bases. The vertical axis (band value) represents the band value corresponding to each off-diagonal, which is the average of all elements on that diagonal. These band values reflect the statistical distribution of relative distances between base pairs in 4D space. The numerical representation method of bandwidth offers high flexibility, simple computation, and time efficiency. It allows for comparing sequences of arbitrary lengths. It is widely used in the field of bioinformatics and computational biology, such as DNA sequence alignment, sequence clustering, and phylogenetic tree construction. This method has advantages such as mathematical clarity, determinism, and independence from the direction of the bases [82]. It provides a quantitative analysis tool for studying the similarity and differences between DNA sequences. However, this method loses the ability of graphical visualization and direct comparison of sequences, making it relatively weaker in terms of sequence visualization and intuitive comparison.

### 5.2. Chemical and Physical Properties Numerical Representation

The numerical representation method of the chemical and physical properties determines the coordinate values of bases using the classification of their chemical properties (R/Y, M/K, W/S) [83]. This method simultaneously considers the physical and chemical structures of the sequence. The specific construction method is as follows: The initial DNA sequence, $S=s_{1},s_{2},s_{3},\cdots s_{N}$, is simplified into a set of points, $\varphi (S)=\varphi (g_{1})\varphi (g_{2})\varphi (g_{3})\cdots \varphi (g_{N})$. The mathematical model corresponding to each point is as follows:

(4) $$\varphi (g_{i})=\left\{\begin{array}{c} (1,1,1,1-\frac{1}{i})\;\mathrm{if}\;g_{i}=A\;\\ (1,0,0,1-\frac{1}{i})\;\mathrm{if}\;g_{i}=G\\ (0,1,0,1-\frac{1}{i})\;\mathrm{if}\;g_{i}=C\\ (0,0,1,1-\frac{1}{i})\;\mathrm{if}\;g_{i}=T \end{array}\right.$$

where i=1,2,3⋯N,N represents the length of the sequence, and the resulting 4D representation of the DNA primary sequence is unique. By performing simple calculations, the geometric center of the base distribution frequencies in the sequence can be obtained [84]. At the same time, we can obtain the relationships between the different bases in the DNA sequence. The geometric center of a sequence is fixed, so it can be used as an invariant in the matrix to compare the similarity between two DNA sequences. This method also avoids information loss during the transition from DNA sequence to mathematical representation [85]. However, a drawback is that it loses the visual representation of the sequence.

### 5.3. Bases Combinations Numerical Representation

The numeric representation method of base combinations utilizes a combination of four nucleotides (A, T, G, C), 16 base pairs, and 64 codons to establish a new representation method [86]. The 4D spatial coordinates of $\varphi \left(g_{i}\right)$ are defined as ($X_{m},\;\;Y_{n},Z_{p},W_{q}$). The value of $X_{m}\;$(m<=N) is determined based on the priority order of individual nucleotides occurring in the sequence, i.e., [3, 2, 1, 0]. $Y_{n}(n$<=N) represents the values associated with the 16 possible combinations of nucleotide pairs. When n<N, the specific calculation of each nucleotide pair is given by equation (5). When the nucleotide pair consists of the N th nucleotide and a space, the value is equal to the average of the values of the four nucleotide pairs formed by the N th nucleotide with the four nucleotides. $Z_{p}$(p<=N) represents the numerical value of the 61 codons encoding amino acids. $W_{q}$ represents the number of occurrences of the four nucleotides (A, T, G, C) in the sequence [87,88].

(5) $$\varphi (g_{i}g_{i+1})=\left\{\begin{array}{c} 1\;\mathrm{if}\;g_{i}g_{i+1}=\mathrm{AA}\;\\ 2\;\mathrm{if}\;g_{i}g_{i+1}=\mathrm{AT}\;\\ 3\;\mathrm{if}\;g_{i}g_{i+1}=\mathrm{AG}\;\\ 4\;\mathrm{if}\;g_{i}g_{i+1}=\mathrm{AC}\;\\ 5\;\mathrm{if}\;g_{i}g_{i+1}=\mathrm{TA}\;\\ 6\;\mathrm{if}\;g_{i}g_{i+1}=\mathrm{TT}\;\\ 7\;\mathrm{if}\;g_{i}g_{i+1}=\mathrm{TG}\;\\ 8\;\mathrm{if}\;g_{i}g_{i+1}=\mathrm{TC}\;\\ 9\;\mathrm{if}\;g_{i}g_{i+1}=\mathrm{GA}\;\\ 10\;\mathrm{if}\;g_{i}g_{i+1}=\mathrm{GT}\;\\ 11\;\mathrm{if}\;g_{i}g_{i+1}=\mathrm{GG}\;\\ 12\;\mathrm{if}\;g_{i}g_{i+1}=\mathrm{GC}\;\\ 13\;\mathrm{if}\;g_{i}g_{i+1}=\mathrm{CA}\;\\ 14\;\mathrm{if}\;g_{i}g_{i+1}=\mathrm{CT}\;\\ 15\;\mathrm{if}\;g_{i}g_{i+1}=\mathrm{CG}\;\\ 16\;\mathrm{if}\;g_{i}g_{i+1}=\mathrm{CC}\; \end{array}\right.$$

Taking a base segment S as an example, the calculation of coordinates in 4D space is shown in Table 1. Based on the coordinate values of the points in 4D space, it can be inferred that there is a one-to-one correspondence between any DNA sequence and its 4D graphical representation [89,90]. This representation fully captures the biological information of the DNA sequence and prevents information loss.

.

By using the 4D coordinates of each nucleotide in a DNA sequence fragment, it is possible to calculate the geometric center of the sequence fragment. The computed geometric center can be used as an invariant feature of the sequence. By calculating the Euclidean distance and the angle between two feature vectors, we can measure the similarity between the two sequences. This numerical representation method based on single, double, and triple base combinations combines the physical and chemical properties of DNA sequences, allowing for an effective representation of the structural features of the sequences [91]. This method has extensive applications in various fields such as DNA sequence alignment, classification, and analysis.

The four-dimensional numerical representation method not only possesses significant computational efficiency advantages but also offers unique insights into evolutionary biology that other visualization methods cannot provide. Firstly, by integrating multi-dimensional attributes, it provides a more comprehensive perspective for revealing similarities and variabilities among sequences. Secondly, it can precisely quantify the similarities and differences of sequences of any length, offering an efficient tool for studying sequence similarities and variabilities. Additionally, it can reveal the distribution characteristics and patterns of base properties within sequences, which aids in analyzing key functional regions or specific pattern changes within the sequences.

## 6. Discussion and Prospect

### 6.1. Analysis of Different Sequence Visualizations

#### 6.1.1. Two-Dimensional Representation

The two-dimensional representation of DNA sequences is used to intuitively visualize and analyze key features of DNA sequences in computational biology. Its primary advantages are as follows [92,93,94]:

(1) Intuitiveness: two-dimensional representation enables quick identification of base distribution patterns and probabilistic features.
(2) Information richness: two-dimensional representation captures and conveys more information than one-dimensional methods, providing a richer display of data.
(3) Handling large data volumes: Two-dimensional representation methods can effectively process large datasets. For example, DNA sequence visualization models based on grayscale images can handle extensive DNA sequences with minimal loss of information.
(4) Low computational complexity: graphical representation methods provide a low computational complexity approach to describing gene sequences, significantly accelerating the research process compared to traditional approaches.

However, the two-dimensional representation of DNA sequences also has several notable drawbacks [95,96]:

(1) Information loss: some two-dimensional models may be unsuitable for representing long DNA sequences, potentially resulting in the loss of detailed information and increasing the risk of observers overlooking critical data.
(2) Limited distinguishability: visualization based on grayscale images may have limited distinguishability, potentially hindering the effective extraction of useful information.
(3) Localization ambiguity: when distribution differences arise in the graphical representation of gene sequences, pinpointing the exact locations of the differing sequences becomes challenging.
(4) Technical limitations: Two-dimensional visualization models, such as spectral-based models, are effective in revealing the properties of shorter DNA sequences. However, they face challenges when applied to long DNA sequences due to limitations in resolution and clarity. In such cases, key structural and compositional features may become obscured or difficult to interpret within a two-dimensional framework.

Despite these limitations, two-dimensional representation methods excel in handling large datasets composed of multiple shorter DNA sequences. Their efficiency and intuitive visualization capabilities make them particularly suitable for analyzing high-volume datasets. Nonetheless, challenges such as potential information loss and limited distinguishability remain [97]. Therefore, selecting an appropriate representation method should be guided by specific research objectives and data characteristics.

#### 6.1.2. Three-Dimensional and Higher-Dimensional Representation Approaches

Three-dimensional DNA sequence representation methods are widely used to capture and analyze the spatial organization and interactions of DNA sequences in three-dimensional space, providing distinct advantages in computational biology [98,99].

(1) Understanding spatial conformation mechanisms: three-dimensional genomics provides a clearer understanding of the mechanisms underlying changes in chromatin spatial conformation and gene transcriptional regulation.
(2) Comprehensive mapping of intracellular chromatin interactions: three-dimensional genomics facilitates the comprehensive mapping of chromatin interactions within cells.
(3) Revealing genome functional mechanisms: three-dimensional genomics, combined with 3C-based techniques and other derivative sequencing technologies, has provided scientists with an increasingly clear understanding of the spatial conformation of genomes in plants, animals, and microorganisms.
(4) Predicting Three-Dimensional Interactions: recent machine learning approaches can predict three-dimensional spatial interactions from one-dimensional epigenomic data or DNA sequences, as shown by models like DeepC and Akita [100,101].

The main drawbacks of three-dimensional and higher-dimensional DNA sequence representation methods include the high complexity of data integration analysis and high computational resource requirements. Higher-dimensional representation methods generate vast and complex datasets, making the integration and analysis of three-dimensional structural differences across different sequence types highly challenging. Predicting three-dimensional structures from one-dimensional sequences demands substantial computational resources to extract meaningful information [102,103].

Higher-dimensional DNA sequence representation methods offer significant advantages for understanding the three-dimensional structure of genomes. However, they also face challenges such as complex data processing, high costs, and limited analysis tools. With continued technological advancements, these limitations are expected to be mitigated, enabling broader and more in-depth applications of multi-dimensional DNA sequence representation in computational biology [104,105].

#### 6.1.3. Influence of DNA Sequence Representation on Machine Learning

(1) Differences in feature extraction capability: DNA sequence representation methods convert sequence data into numerical features that can be processed by machine learning models. Different representation methods capture various local features (such as domains or motifs) and global features (such as information from entire coding regions), thereby influencing the performance of machine learning models.
(2) Differences in computational complexity: two-dimensional and higher-dimensional sequence representation methods vary in data volume, storage requirements, and modeling mechanisms within machine learning models, resulting in differences in computational complexity.
(3) Differences in machine model selection: model selection is often guided by the specific DNA sequence representation method used, as different models have varying requirements for DNA sequence representations.
(4) Differences in model generalization ability: Different DNA sequence representation methods exhibit varying levels of generalization ability. Due to differences in the underlying mechanisms of these representations, machine learning models demonstrate different adaptability to sparse or imbalanced training data. Low-dimensional representations generally offer better generalization than higher-dimensional representations.

DNA sequence representation methods impact various aspects of machine learning models [106,107], including feature extraction, model performance, computational complexity, prediction accuracy, and generalization ability [108,109,110]. Therefore, selecting an appropriate DNA sequence representation method is essential for building effective machine learning models.

### 6.2. Application Fields of Sequence Visualization

#### 6.2.1. Application Field Overview

DNA sequence visualization is not only a tool for displaying genetic information but also a crucial method for analyzing biological data and decoding complex biological phenomena [111,112]. Ensemble learning models that integrate DNA visualization with machine learning hold especially significant value in the field of biology. DNA visualization not only provides the model with rich input information but also allows for an intuitive display of output results, thereby enhancing the model’s accuracy, interpretability, and practical effectiveness. Such ensemble learning models demonstrate broad applicability and value in the fields of biology and medicine [92,113].

(1) Genome Analysis and Disease Decoding

In genome analysis and disease decoding, DNA sequence visualization can integrate information from multiple data sources, such as genomic sequences, transcriptomic data, protein interaction networks, and epigenetic information. By using these multi-source data as inputs for machine learning models, researchers can effectively identify structural variations in chromosomal regions and gene regulatory networks. Presenting these complex data relationships in a visual format allows machine learning models to better reveal associations between genetic variations and diseases, offering new perspectives for studying disease mechanisms.

(2) Personalized Medicine

In the field of personalized medicine, DNA sequence visualization combined with machine learning offers new possibilities for developing precise medical treatments. This approach shows significant value, particularly in gene mutation prediction and the design of gene editing and targeted therapies. By visually presenting machine learning predictions, researchers can intuitively interpret a patient’s specific mutations, providing strong support for the development of personalized diagnostic and treatment plans.

(3) Gene Expression and Regulatory Prediction

In gene expression and regulatory prediction, machine learning models (such as BigRNA) can predict regulatory features from DNA sequences, including RNA expression levels, splice sites, and microRNA binding sites. By visualizing these predictions, researchers can identify key non-coding variants and regulatory regions, further exploring their roles in gene expression regulation and their potential impact on disease.

(4) Promoter Engineering Research

In promoter engineering, visualization combined with machine learning models can be used to build quantitative models of promoter sequences. This approach allows for the effective analysis of their functional characteristics. The visualization of promoter sequences can reveal the regulatory effects of different sequences on gene expression. This provides scientists with data support for designing promoters to meet specific expression needs, enabling the precise control of gene expression.

(5) Genomics Research

In genomics research, DNA sequence visualization combined with machine learning models is used to predict gene regulatory networks, identify essential genes, and analyze complex genome structures. By using DNA visualization for multi-dimensional data integration and analysis, researchers can gain deeper insights into the relationships between genes and phenotypes, thereby advancing progress in genomics research.

#### 6.2.2. Multidimensional Biological Data Integration and Analysis

Multidimensional omics data, including genomics, transcriptomics, proteomics, and metabolomics, have been increasing exponentially [114,115]. The integration and analysis of these diverse biological datasets have become essential for understanding complex biological systems. DNA sequence visualization techniques play a crucial role in this field, facilitating the systematic integration, data mining, and feature analysis of various omics datasets [116,117]. These techniques enable researchers to understand the functions and regulatory mechanisms of complex biological networks at multiple levels. Integrative analysis enables a more comprehensive elucidation of the interactions and dynamic changes in various biological information at the cellular, tissue, or organismal levels [118,119].

(1) Heterogeneous Integration and Standardization of Multi-Omics Data

The integration of multi-omics data is a major research focus in bioinformatics. With the rapid accumulation of multidimensional omics datasets, integrating these heterogeneous data to uncover the complex regulatory networks within biological systems has become a critical challenge. However, traditional sequence numerical encoding faces notable limitations in multi-omics data integration. These limitations primarily include inconsistent expression standards across different data sources, complex quantification methods, and considerable variability in sequence lengths. Consequently, numerical encoding often results in inconsistencies in data dimensionality, which further complicates feature extraction and increases model processing complexity.

DNA sequence visualization techniques provide an effective solution for multi-omics data integration. Through visual encoding, heterogeneous data such as genomics and transcriptomics can be intuitively displayed and compared on a unified platform, ensuring consistency in data format and scale. Sequence visualization not only highlights the key features of each omics data type but also effectively reveals inter-layer relationships, facilitating cross-platform integration of multi-omics data. Additionally, sequence visualization assists in identifying and correcting outliers or biases, reducing error accumulation stemming from format discrepancies. This integration and standardization process enhances data comparability and consistency, establishing a robust foundation for multi-omics integrative analysis.

(2) Complex Feature Extraction from High-Dimensional Sequence Data

Feature extraction is essential in high-dimensional sequence data analysis. However, the context-dependent features commonly found in sequence data are often difficult to capture accurately through numerical encoding methods. Numerical encoding not only fails to represent the complex relationships and regulatory information between sequences but also, due to its high dimensionality, results in incomplete feature extraction and introduces redundant features. This encoding approach further increases the time complexity and computational cost of data processing, ultimately diminishing the model’s accuracy and efficiency.

Transforming high-dimensional sequence data into graph structures is an effective strategy for feature extraction. Graph structures can flexibly accommodate sequence data of varying lengths and extract multidimensional features from inter-sequence relationships. For instance, dynamic visualization techniques can illustrate temporal changes in gene expression, DNA mutations, and metabolomic features, making them particularly suited for studying processes like cell differentiation and drug response, and revealing the temporal dependencies and dynamic mechanisms underlying these biological phenomena. Graph structures are highly effective at capturing spatial relationships, functional modules, and other biologically relevant information within high-dimensional data, thereby enhancing the biological significance of extracted features. Additionally, through network analysis and clustering methods, graph structures can effectively reduce redundant features, improving both the representativeness and discriminative power of the features. This multidimensional feature extraction approach not only simplifies the processing of high-dimensional data but also significantly enhances data interpretability, providing a solid foundation for subsequent data analysis and biological research.

#### 6.2.3. Disease-Associated Mutation Analysis and Personalized Therapy

(1) Mutation Prediction in Personalized Medicine

Predicting gene mutations is essential for personalized medicine. Accurate mutation prediction assists in assessing genetic disease risk, optimizing drug response, and developing targeted treatment plans, thus providing scientific support for early diagnosis and personalized therapeutic strategies. However, gene mutation data originate from diverse individuals, with variations in mutation locations and types causing inconsistencies in data dimensionality and scale [120,121]. Conventional numerical encoding methods struggle to flexibly adapt to these variations, often resulting in information loss or error accumulation, which subsequently impacts the accuracy and stability of mutation predictions. Moreover, the complex characteristics of gene mutations depend not only on sequence context but also on spatial and regulatory interactions with other genes and regulatory elements—interactions that numerical encoding fails to capture effectively.

To address the limitations of numerical encoding, DNA sequence visualization techniques offer an efficient solution. By visualizing mutations and polymorphisms, the complex information within gene mutations can be intuitively displayed, making their distribution, frequency, and associations with diseases easily discernible. Tools such as heatmaps, distribution plots, and 3D structural visualizations enable researchers to rapidly identify mutation hotspots, rare variants, and regions with high-frequency polymorphisms, thereby uncovering potential biological functions. The intuitive nature of visualization techniques enhances data interpretability, allowing clinicians to analyze and interpret mutation data more effectively and providing strong support for mutation analysis in personalized medicine [122,123,124].

(2) Gene Editing and Targeted Therapy Design

In gene editing and targeted therapy design, DNA sequence visualization techniques provide essential support for target identification and mutation repair design. By leveraging 3D visualization to reveal the spatial structure of mutation sites within the genome, researchers can precisely locate editing targets and surrounding regulatory regions, thereby enhancing the accuracy and efficiency of gene editing [125,126]. This visualization approach not only aids in optimizing gene editing design and minimizing off-target effects but also ensures the specific impact of editing operations on the target region, laying a robust technical foundation for the advancement of precision medicine and targeted therapies [127,128].

### 6.3. Potential Research Directions

Traditional DNA sequence visualization methods have limitations when dealing with long sequences, mainly manifested as the loss of global structure and local details due to information density, high complexity in data integration, and the significant consumption of computational resources. To address these issues, this paper proposes two potential approaches: Firstly, the construction of a knowledge graph for DNA sequence big data is proposed, which integrates genome data; annotates functional regions, mutation hotspots, and sequence patterns; and combines high-dimensional visualization techniques to achieve the precise parsing and efficient display of long sequences. Secondly, the integration of machine learning with DNA sequence visualization is suggested, which through feature extraction and pattern recognition, automatically identifies important patterns and functional areas, reduces redundant information, and generates concise and meaningful visualization images, providing an efficient solution for the analysis of long sequences.

#### 6.3.1. Construction of Knowledge Graph for DNA Sequence Big Data

Biological sequence data exhibit characteristics such as being sourced from multiple sources, having heterogeneity, and being loosely organized [129]. There are many challenges in extracting knowledge from massive biological data. Biologists often use specialized terms, such as SNOMED, CT, ICD-10, PubChem, Gene Ontology (GO), and so on [130,131]. These terms convey important semantics and consensus among biologists. However, for computer experts, these terms are often dry and obscure, making them difficult to understand [132,133]. Especially when dealing with large-scale data, it becomes challenging to precisely locate the required knowledge. When biological researchers use computer programs or software for bioinformatics analysis, the time complexity is often high. Moreover, they need to filter out potentially useful data from numerous search results, and the process of handling long sequences of genes and genomes is very time-consuming [134,135]. Therefore, traditional computer programs have limitations in retrieving and analyzing sequencing data, and they cannot meet the scientific community’s needs for information connectivity and search. To address this issue, establishing a language that can be understood by biologists, computer scientists, and machines alike through the use of machines and machine learning is a fundamental solution [136].

The advancement of sequencing technologies has a major advantage in providing low-cost digital data and high-resolution sequencing results. This is a prerequisite for knowledge extraction. When it comes to storing large-scale sequencing data, there is a growing focus on building more efficient data structures and management systems [137,138]. Indeed, data are not equivalent to knowledge, so further exploration is needed on how to perform optimal searches on data to extract meaningful knowledge from them. To be exact, the development of suitable analysis software and integrated platforms is crucial to better establish a semantic network of data.

Knowledge graphs (KGs) are a technique that describes concepts, entities, and their relationships in the objective world in a structured manner [139,140]. It can represent information on the Internet in a form that is closer to the human cognitive world, providing a better ability to organize, manage, and understand the vast amount of information on the Internet [141,142,143,144]. On one hand, they can be continuously updated with new knowledge and information. On the other hand, knowledge may be distributed across different data sources. KGs play a significant role in addressing the challenges of biological sequence big data [145]. By utilizing KGs, it becomes possible to establish richer semantic networks within sequencing data by linking them with other biological knowledge and entities. By leveraging inferences and querying on the concepts and relationships within the KG, it is possible to achieve semantic search and knowledge extraction from sequencing data [146,147]. Therefore, establishing a graphical association KG system for biological sequences is an important direction of development.

(1) General framework

**Figure 15:**
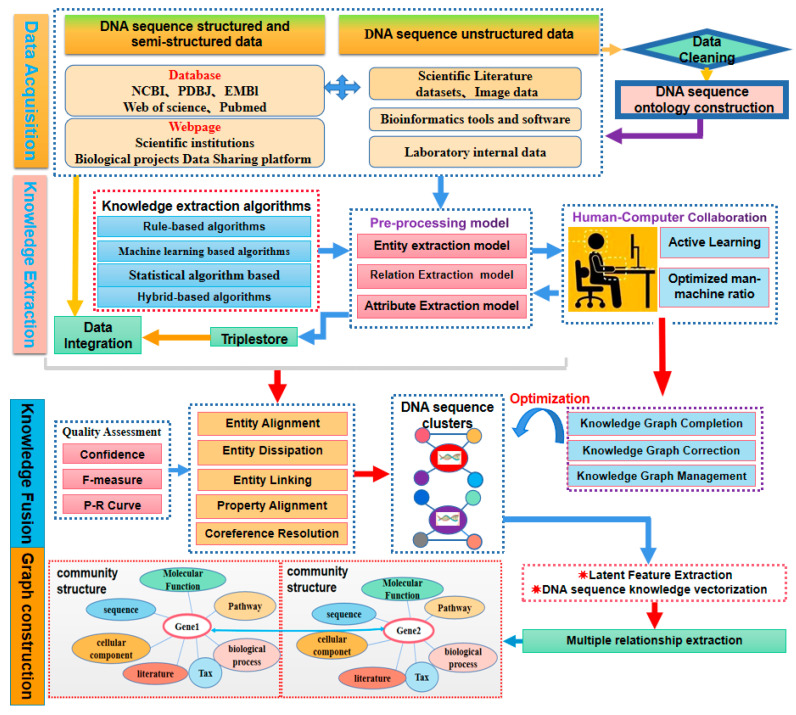
Framework diagram for building a knowledge graph of DNA sequences.

As an opening for further discussion and exploration, this article proposes a concept and research directions for constructing a KG of DNA sequence big data by utilizing KG-related technologies and focusing on the characteristics of DNA sequence big data [137,148]. The aim is to provide researchers with a new solution for the in-depth exploration and decision analysis of massive DNA sequence data [149,150]. The framework and key technologies for constructing KGs are shown in Figure 15.

(2) Ontology Construction

**Figure 16:**
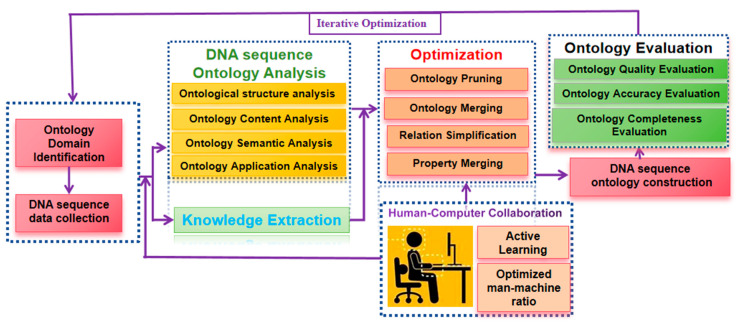
Framework for DNA sequence ontology construction.

To ensure the reliability of entity recognition results in biological sequence, it is necessary to construct a DNA sequence ontology library. It is recommended to adopt a combined approach of top-down and bottom-up methods to construct the DNA sequence ontology library [151,152]. The framework of ontology construction is illustrated in Figure 16.

Firstly, it is to determine the professional domain and application scope of the DNA sequence ontology. For the DNA sequence KG, the ontology is oriented towards the fields of DNA sequence function, structure, origin, organization, biological processes, and sequence variations. The scope of application involves the semantic description and inference capabilities of a DNA sequence. It is used to support applications such as data management, data mining, knowledge discovery, and knowledge inference in the fields of bioinformatics, genomics, biomedical research, and other related areas [153,154]. Secondly, DNA sequence data collection should be carried out based on the identified professional domains and application scope. Thirdly, a collaborative approach involving both human experts and machine analysis should be employed to extract the key terms for ontology from the data sources [155]. At the same time, the entities obtained in the subsequent knowledge extraction stage should also be included as key terms in the ontology [156,157,158]. Fourth, ontology optimization and simplification should be conducted, also using a collaborative approach involving both human expertise and computational methods [159,160,161]. Fifth, the DNA sequence ontology should be constructed and evaluated using three metrics. Sixth, the professional domain and application scope of the DNA sequence ontology should be iteratively optimized based on evaluation metrics, and then the above steps should be repeated until the requirements are met [162,163,164].

(3) Community structure fusion

Community structure can be seen as a network structure composed of different “clusters”. Within each “cluster”, the nodes are more densely connected, while the connections between nodes from different “clusters” are sparser. In the construction of a DNA sequence big data KG, genes with similar functions in different species may form distinct “clusters” [149]. For example, the first exon sequences of the human β-globin gene and the first exon sequences of the gorilla β-globin gene form two different “clusters”. How to establish connections between these different “clusters” and form a community structure is an important question. Furthermore, the identification of existing sequence community structures, the acquisition and querying of community data, as well as the analysis of the overall network structure and functionality have important implications for gene function prediction, essential gene identification, and other related aspects [165,166]. However, traditional community detection algorithms typically only consider the structural characteristics of the network, lacking the necessary considerations for semantic information within the KG.

(4) Knowledge fusion

**Figure 17:**
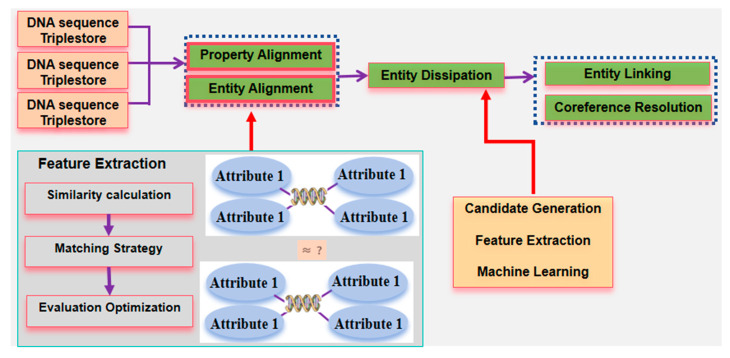
Knowledge fusion framework for DNA sequence.

Different biological knowledge bases use different entity identifier systems, which can lead to ambiguity when merging entities in a consolidated biological database [167]. When integrating heterogeneous data, typical problems include entity alignment, attribute alignment, entity disambiguation, entity linking, and co-reference resolution [168,169]. The knowledge fusion framework is illustrated in Figure 17.

DNA sequence entity alignment is a core step in knowledge fusion. First, extract entity data features from DNA sequence triplets, such as attribute values, labels, descriptions, relationships, etc., as input for entity alignment [170]. Based on the obtained entity features, calculate the similarity or distance between entities (entity attribute similarity measure). Common similarity calculation methods include cosine similarity, edit distance, Jaccard similarity, etc. Secondly, entity alignment is performed based on the calculated entity similarity [24]. Different matching strategies can be used, such as a threshold-based strategy, similarity ranking strategy, machine learning methods, etc. Finally, the entity alignment results are evaluated using metrics such as precision, recall, F1 score, etc. [171,172]. Based on the evaluation results, the entity alignment can be optimized and adjusted.

Entity disambiguation is performed after entity alignment and attribute alignment. First, generate a set of possible entity candidates. Secondly, extract features from the entity candidate set and contextual information [173]. Finally, use machine learning or rule-based methods to calculate the weights or probabilities of the entity candidate set based on the extracted features, and choose the most likely entity as the disambiguation result [174].

After entity disambiguation, entity linking and coreference resolution are performed. The process of entity linking mainly involves two steps: (1) candidate entity generation; (2) candidate entity ranking. After entity linking, co-reference resolution is performed to improve the accuracy of entity linking.

(5) Named Entity Recognition (NER)

**Figure 18:**
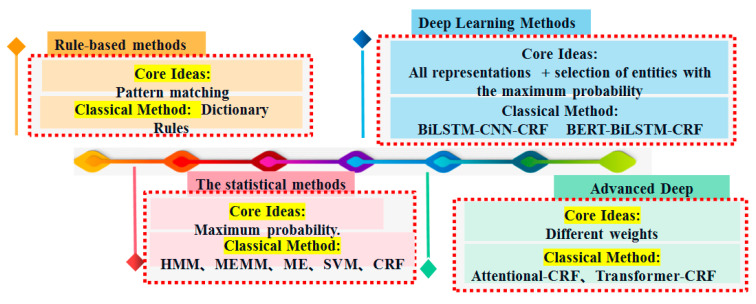
Named entity recognition methods for DNA sequence.

Entities are an essential component in building KGs. The accuracy of entity recognition is crucial for building KGs [175,176]. The data sources for constructing a KG of DNA sequence big data include structured, unstructured, and semi-structured data such as research papers, biological software and tools, and image data [177,178]. Therefore, performing entity recognition from these vast amounts of textual and image data is a crucial task for constructing a KG. The NER for a DNA sequence is illustrated in Figure 18.

Currently, commonly used entity recognition methods include rule-based entity recognition, statistical machine learning-based entity recognition, and deep learning-based entity recognition, among others [179,180]. These methods utilize specific patterns, statistical models, or deep neural networks to extract entities from text or image data, thereby extracting the necessary entity information for constructing a KG [181].

#### 6.3.2. Machine Learning-Based DNA Sequence Image Generation

There is a lack of unified standards for visualizing DNA sequences. Different methods are used to visualize different DNA sequences, and different features are selected for visualization representation using different methods [182,183,184,185]. For example, some methods focus on global features, while others focus on local features, resulting in generated graphics with different emphases, and may not provide sufficient semantic information to meet the requirements of customized views for biological sequences [186,187,188]. This limitation hinders the extension and application of knowledge. Therefore, building image generation models that transform DNA sequence textual descriptions into DNA sequence images that reflect the textual information is an important task.

The current mainstream generation models for addressing the text-to-image generation task include auto-regressive models, variational autoencoders, and Generative Adversarial Networks (GANs) [189,190,191]. An AM generates images by progressively generating pixels or local features of the image step by step. A VA generates new samples by learning the latent space of the data. The GAN has become the most popular method due to its powerful sample generation capability. It continuously improves the quality of generated images through adversarial training between the generator and discriminator. Therefore, there is a wide range of promotion and application value in generating DNA sequence images using advanced machine learning algorithms.

(1) DNA sequence image generation framework

**Figure 19:**
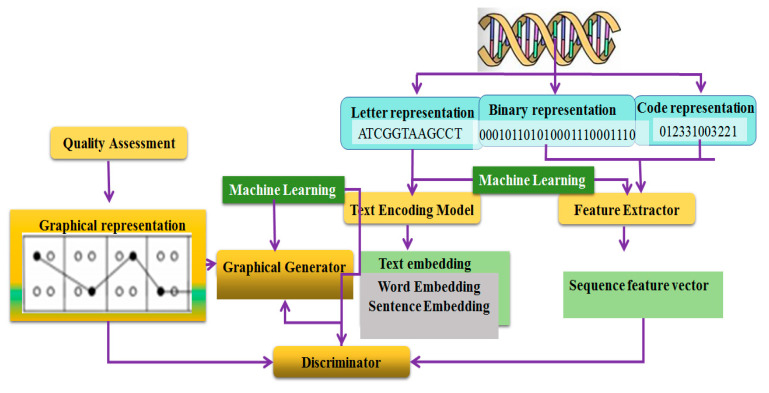
Machine learning-based framework for DNA sequence image generation.

As an opening for further discussion and exploration, this review combines the features of DNA sequences and leverages the advantages of machine learning algorithms to propose a research idea for constructing visual representations of DNA sequences using machine learning methods [192]. The purpose of this idea is to provide researchers in interdisciplinary fields such as biology and computer science with a new perspective on handling and analyzing large-scale DNA sequence data [193,194]. This research perspective holds the potential to provide a new method for constructing high-quality image generation models in DNA sequence visualization. By leveraging machine learning techniques and considering the specific requirements of different application scenarios, it aims to address the need for customizable views of biological sequences [195,196]. The image generation framework and key techniques are illustrated in Figure 19.

First, the DNA sequence structure is represented through textual and numerical descriptions. Secondly, a machine learning algorithm is employed to construct a text encoding model, which generates text embedding vectors (such as word embeddings and sentence embeddings) through training [197]. Simultaneously, a machine learning model is used to construct a feature extractor for numerical descriptions, which extracts numerical features from the sequence. Third, a machine learning pre-trained image generator is employed to generate draft images using the text embedding vectors as input. Fourth, a machine learning algorithm is employed to pre-train the discriminator. The sequence’s numerical feature is then associated with the generated draft image to provide fine-grained supervisory feedback, thereby facilitating the training of an effective image generator. Finally, the generated images are subjected to quality evaluation [198].

(2) Machine Learning Algorithms in DNA Sequence Image Generation

**Figure 20:**
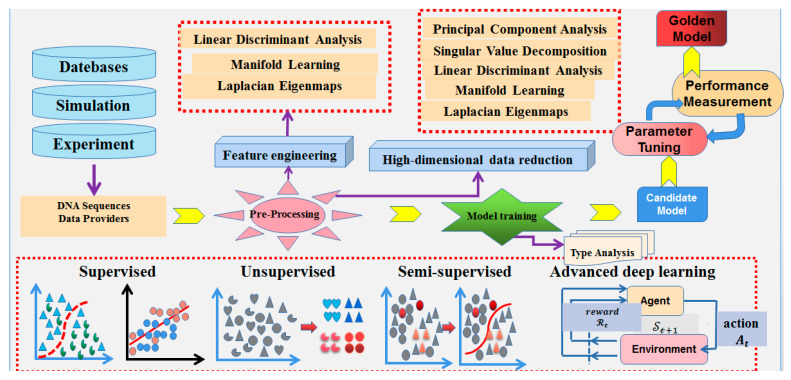
Machine learning algorithms for DNA sequence image generation.

The textual description of DNA sequences is stored as a one-dimensional sequence, while DNA sequence images are stored in the form of two-dimensional structural information. Therefore, there is a significant difference between textual description and image representation in terms of feature representation and information expression [199,200]. This difference can potentially lead to issues such as the generated image having an unreasonable layout and being inconsistent with the textual description. Therefore, when selecting algorithms, it is necessary to consider the differences in feature representation and information expression between text and images and to choose suitable algorithms to generate DNA sequence images with a reasonable layout and accurate representation. There are currently various machine learning algorithms available, but there is no single algorithm that is optimal for all tasks [201,202]. The main machine learning algorithms to generate DNA sequence images are depicted in Figure 20.

The entire process of machine learning includes steps such as analyzing objectives, collecting data, organizing data, preprocessing data, training models, evaluating models, and optimizing models [203,204,205]. Depending on the type of data collected, different types of machine learning algorithms can be chosen for model training. Based on learning methods, the main types of machine learning algorithms are supervised learning, unsupervised learning, semi-supervised learning, and advanced deep learning [206,207,208,209].

Supervised learning: Training is performed using input data along with their corresponding labels or target values. The goal is to establish a mapping relationship between the input and the output [210,211]. By learning from known input–output data, the model can predict the output for new, unseen data. It mainly includes decision trees, support vector machines, Bayesian networks, neural networks, and ensemble learning methods such as random forests. Unsupervised learning: By learning the internal structure and relationships of the input data, it is possible to discover patterns, categories, or other structural information within the data [212]. Unlike supervised learning, unsupervised learning does not have explicit labels or target values to guide the learning process. It mainly includes clustering algorithms, principal component analysis (PCA), and association rule mining, among others. Semi-supervised learning: Semi-supervised learning algorithms are a learning approach that falls between supervised learning and unsupervised learning. It utilizes both labeled training data and unlabeled training data to learn, aiming to improve learning performance and generalization by leveraging information from both labeled and unlabeled data [213]. Semi-supervised learning algorithms are often applied in situations where labeled data are difficult to obtain. Advanced deep learning: Advanced deep learning refers to more complex and advanced techniques and methods within the field of deep learning. It builds upon the foundation of basic deep learning models and algorithms. It involves the use of deeper and more complex neural network architectures, as well as more advanced training techniques, to tackle more challenging and intricate problems. Some commonly used advanced deep learning methods include Convolutional Neural Networks (CNNs), Recurrent Neural Networks (RNNs), Advanced Recurrent Neural Networks, Deep Belief Networks (DBNs), Generative Adversarial Networks, Reinforcement Learning (RL) [214,215], and so on. These different types of machine learning algorithms are suitable for various problems and scenarios. Choosing the appropriate learning algorithm based on specific task requirements and data characteristics can help achieve better learning and prediction performance.

## 7. Summary

This article presents a comprehensive review of DNA sequence visualization methods based on two-dimensional, three-dimensional, four-dimensional, and dynamic visualization. Additionally, this article discusses and analyzes the strengths and limitations of each method. Furthermore, this article introduces the applications of visualization methods in sequence similarity measurement, gene mutation analysis, and homology analysis. It analyzes the advantages and limitations of these applications. In addition, this article proposes two potential research directions: the construction of a KG for DNA sequence big data and the application of machine learning methods for DNA sequence image generation. Visual methods play a crucial role in DNA sequence analysis, but they also have some limitations. For example, there are limitations in terms of visualization effectiveness, data capacity for visualization, and high algorithmic complexity. Future research directions include improving visualization effectiveness, enhancing the processing capabilities of visualization techniques, and increasing the interactivity of data visualization. These advancements aim to better meet the requirements of DNA sequence analysis. This article contributes to the comparison and evaluation of research from different disciplines, promoting the improvement and development of visualization methods for biological sequences.

## Figures and Tables

**Table 1:** Four-dimensional coordinate values of each base in sequence fragment S=ATGGTGCACC.

Single Nucleotide	$X_{m}$	Base Combinations	$Y_{n}$	Triplet Bases	$Z_{p}$	$W_{q}$	4D Coordinates
A	3	AT	2	ATG	−2	1	(3,2,−2,1)
T	2	TG	7	TGG	1	1	(2,7,1,1)
G	1	GG	11	GGT	0	1	(1,11,0,1)
G	1	GT	10	GTG	−2	2	(1,10,−2,2)
T	2	TG	7	TGC	0	2	(2,7,0,2)
G	1	GC	12	GCA	−2	3	(1,12,2,3)
C	0	CA	13	CAC (ACA + ACG + ACU + ACC)	2	1	(0,13,2,1)
A	3	AC (CA + CU + CC + CG)	4	ACC	1	2	(3,4,1,2)
C	0	CC	16	CC_	0	2	(0,16,0,2)
C	0	C_	14.5	C__	0	3	(0,14.5,0,3)

## Data Availability

No new data were created or analyzed in this study.

## Abbreviation

Abbreviation	Detail
Attentional-CRF	Attentional CRF
BERT	Bidirectional Encoder Representations from Transformers
BiLSTM	Bidirectional Long Short-Term Memory
BIRCH	Balanced Iterative Reducing and Clustering Using Hierarchies
BN	Bayesian Network
CGR	Chaos Game visualization
CMI	Coded Mark Inversion
CNN	Convolutional Neural Network
CRF	Conditional Random Field
DBN	Deep Belief Network
DBSCAN	Density-Based Spatial Clustering of Applications with Noise
DNA SV	DNA sequence visualization
DN-curve	Double Nucleotide Curve
DT	Decision Tree
DV-Curve	Double Vector
EA	Evolutionary Algorithm
GANs	Generative Adversarial Networks
HMM	Hidden Markov Model
KG	Knowledge Graph
LSR	Letter Sequence Representation
LSTM	Long Short-Term Memory
ME	Maximum Entropy
MEMM	Maximum Entropy Markov Model
NER	Named Entity Recognition
NN	Neural Network
PCA	Principal Component Analysis
RL	Reinforcement Learning
RNNs	Recurrent Neuron Networks
SVM	Support Vector Machine
Transformer-CRF	Transformer with Conditional Random Field
*δ*_1_	ATGGTGCACCTGACTCCTGAGGAGAAGTCTGCCGTTACTGCCCTGTGGGGCAAGGTGAACGTGGATGAAGTTGGTGGTGAGGCCCTGGGCAG
*δ*_2_	ATGGTGCACCTGACTCCTGAGGAGAAGTCTGCCGTTACTGCCCTGTGGGGCAAGGTGAACGTGGATGAAGTTGGTGGTGAGGCCCTGGGCAGG
*δ*_3_	ATGACTTTGCTGAGTGCTGAGGAGAATGCTCATGTCACCTCTCTGTGGGGCAAGGTGGATGTAGAGAAAGTTGGTGGCGAGGCCTTGGGCAG
*δ*_4_	ATGGTGCACCTGACTGATGCTGAGAAGTCTGCTGTCTCTTGCCTGTGGGCAAAGGTGAACCCCGATGAAGTTGGTGGTGAGGCCCTGGGCAGG
*δ*_5_	ATGGTGCACCTAACTGATGCTGAGAAGGCTACTGTTAGTGGCCTGTGGGGAAAGGTGAACCCTGATAATGTTGGCGCTGAGGCCCTGGGCAG
*δ*_6_	ATGCTGACTGCTGAGGAGAAGGCTGCCGTCACCGGCTTCTGGGGCAAGGTGAAAGTGGATGAAGTTGGTGCTGAGGCCCTGGGCAG
*δ*_7_	ATGGTGCACTTGACTTCTGAGGAGAAGAACTGCATCACTACCATCTGGTCTAAGGTGCAGGTTGACCAGACTGGTGGTGAGGCCCTTGGCAG
*ρ*_1_	ACATTTGCTTCTGACACAACTGTGTTCACTAGCACCTCAAACAGACACCATGGTGCACCTGACTCCTGAGGAGAAGTCTGCCGTTACTGCCCGTGGGGCAAGGTGAACGTGGATGAAGTTGGTGGTGAGGCCCTGGGCAGGCTGCTGGTGGTCTACCCTTGGACCCAGAGGTTCTTTGAGTCCTTTGGGGATCTGTCCACTCCTGATGCTGTTATGGGCAACCCTAAGGTGAAGGCTCATGGCAAGAAAGTGCTCGGTGCCTTTAGTGATGGCCTGGCTCACTGGACAACCTCAAGGGCACCTTTGCCACACTGAGTGAGCTGCACTGTGCAAGCTGCACGTGGATCCTGAGAACTTCAAGCTCCTGGGCAATGTGCTGGTCTGTGTGCTGGCCCATCACTTTGGCAAAG
*ρ*_2_	ATGGTGCACTTGACTTCTGAGGAGAAGAACTGCATCACTACCATCTGGTCTAAGGTGCAGGTTGACCAGACTGGTGGTGAGGCCCTTGGCAGGATGCTCGTTGTCTACCCCTGGACCACCAGGTTTTTTGGGAGCTTTGGTGATCTGTCCTCTCCTGGCGCTGTCATGTCAAATTCTAAGGTTCAAGCCCATGGTGCTAAGGTGTTGACCTCCTTCGGTGAAGCAGTCAAGCATTTGGACAACCTGAAGGGTACTTATGCCAAGTTGAGTGAGCTCCACTGTGACAAGCTGCATGTGGACCCTGAGAACTTCAAGATGCTGGGGAATATCATTGTGATCTGCCTGGCTGAGCACTTTGGCAAGGATTTTACTCCTGAATGTCAGGTTGCTTGGCAGAAGCTCGTGGCTGGAGTTGCCCATGCCCTGGCCCACAAGTACCACTAA
φ	CTAACTTTCCTCATTGCTAACCAGCATTCTCATGTCACCTCTCTGTCCTTCAAGCTTCATGTATATAAACTTTCTTTCCAGCCCTTGGGCAT
𝒮	ATGGTGCACC
𝒮′	ATGGTGCACC
𝒮″	ATGCTGACTG
γ	ATGGTGCACCTGACTCCTGA
ℒ′	ATGATGCACC
ℋ	TGTGCTGAGTGAGGTCAGAAACTT
𝒥	ATGACTTTGCTGAGT
